# Residential indoor temperatures and health: A scoping review of observational studies

**DOI:** 10.1016/j.scitotenv.2025.179377

**Published:** 2025-04-27

**Authors:** Janelle R. Edwards, Anneclaire J. De Roos, Chima C. Hampo, Wanyu Huang, Emily Lincoln, Simi Hoque, Leah H. Schinasi

**Affiliations:** a Drexel University, Dornsife School of Public Health, Department of Environmental and Occupational Health, Philadelphia, PA, USA; b Drexel University, Department of Civil, Architectural, and Environmental Engineering, Philadelphia, PA, USA; c Drexel University, Dornsife School of Public Health, Department of Epidemiology and Biostatistics, Philadelphia, PA, USA; d Drexel University, Dornsife School of Public Health, Urban Health Collaborative, Philadelphia, PA, USA

**Keywords:** Residential environments, Indoor temperature, Heat, Climate change, Housing, Mortality, Morbidity, Safe thresholds, Epidemiology

## Abstract

Adults spend most of their time indoors, especially in higher income countries. Indoor temperature exposures can vary substantially across households, even within a single geographic area. It is therefore critical to understand links between indoor temperature exposures and health or well-being outcomes, and to understand safe maximum indoor residential temperature thresholds that support health, well-being, and comfort. We systematically identified peer-reviewed, observational studies that quantified associations between residential indoor temperatures and mortality/morbidity outcomes. We extracted information on study location; population, health or well-being outcomes; indoor temperature exposure assessment methods; and, when available, empirically quantified safe maximum indoor temperature thresholds. In total, 29 papers were included in the review. The studies were conducted in the following continents: North America (*N* = 10), Europe (*N* = 5), Asia (*N* = 9), Australia (*N* = 4), and Africa (N = 1). The most common outcomes were cardiovascular morbidity (N = 10) and respiratory morbidity (*N* = 8) and thermal comfort (*N* = 9). Exposure assessment methods included data sensors, thermometers, data-driven models, and energy-based simulations. Despite variation in exposure assessment methods and outcomes assessed, results predominately suggested that warmer indoor temperatures were associated with adverse health or well-being outcomes, although in a handful of studies, associations were either null or in the unexpected, protective direction. Empirically identified safe thresholds for indoor temperature ranged from 18 °C to 35 °C and varied according to outcome. Results from this review may be used to inform the design of future studies of associations between indoor temperatures and morbidity or mortality outcomes.

## GRAPHICAL ABSTRACT

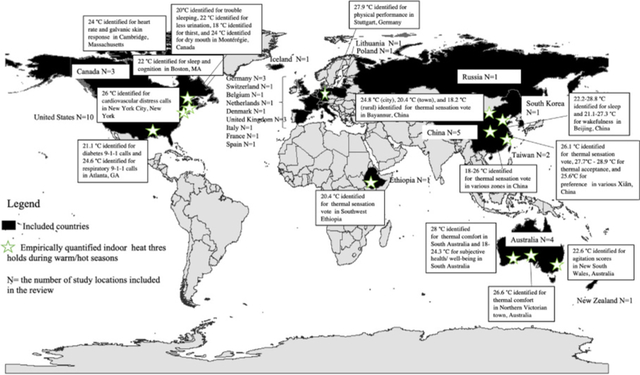

## 1. Introduction

It is well recognized that the earth’s rising temperatures represent a critical threat to population health and well-being (Ebi et al., 2021). It is also well known that most adults living in higher income countries spend nearly all their time indoors (Klepeis et al., 2001; Centers for Disease, 1994). This may be particularly true for heat-sensitive subpopulations, such as older adults, individuals with disabilities or underlying chronic conditions, or those taking medications that enhance dehydration and heat sensitivity (Benmarhnia et al., 2015; Layton et al., 2020; Balbus et al., 2009). It is also well documented that most heat-related deaths occur when people are at home (Fouillet et al., 2006; Centers for Disease, 1994). Yet, to date, nearly all population health studies of associations between ambient heat and adverse outcomes have estimated exposures using outdoor temperature or humidity values (Hajat and Kosatky, 2010; Zanobetti and O’Neill, 2018), often measured at stationary monitors that are not necessarily located near people’s residential addresses.

These current research practices may hamper full understanding of links between high ambient temperatures and population health outcomes. Indeed, the relationship between indoor and outdoor temperature or humidity values is complex, not always linear, and can vary according to season, proximity of a geographic location to the equator, and air conditioning use (Nguyen et al., 2014; Lee and Lee, 2015). In some contexts, indoor and outdoor temperatures have been shown to be strongly correlated, especially when outdoor temperatures are high (Nguyen et al., 2014). However, indoor temperatures have also been found to vary across households, even within the same geographic area (Waugh et al., 2021; White-Newsome et al., 2012; Tamerius et al., 2013). For example, one study, conducted in Greater Boston, Massachusetts, found that summertime indoor temperatures ranged from 10 °C cooler to 10 °C hotter than outdoor temperatures (Nguyen et al., 2014). In Detroit, Michigan, maximum indoor temperatures varied substantially across households, even when restricted to homes with central air conditioning, (White-Newsome et al., 2012). Nevertheless, few population health studies on heat exposure have accounted for intra-household heterogeneity in temperature exposures, potentially leading to biased estimates of association due to exposure misclassification. There is a critical need to improve understanding of links between indoor temperature exposures and health or well-being outcomes in residential settings (Zhang et al., 2019; Wolkoff et al., 2021).

The lack of research on links between indoor temperatures and mortality/morbidity outcomes has also resulted in a lack of clarity about safe maximum thresholds for indoor temperatures. Understanding thresholds is critical for informing messaging about thermostat settings, and for establishing regulations for building design and construction to ensure safe indoor environments (Kenny et al., 2019; Larsen et al., 2023; Anderson et al., 2013; Tham et al., 2020). In addition, it has important policy implications. In some cities, there are laws that require landlords to provide adequate heating to support minimum air temperatures during the cold months of the year (Toronto Municipal Code Chapter 497, 2018; Property Club, 2024; City and County of San Francisco, 2022). However, to date, very few jurisdictions have established policies related to indoor climate control during the hot seasons (Merali, 2023; Toronto Municipal Code Chapter 497, 2018; Montgomery County, Maryland: Bill 24–19, 2020; City of Tempe: Thermal environment, 2024; City of Dallas: SEX 27.1). Strong empirical data on optimal upper thresholds for temperature is needed to inform the creation of policies for healthy residential indoor temperatures.

Currently, global organizations, including the American Society of Heating, Refrigeration and Air Conditioning Engineers (ASHRAE) (ASHRAE, 2023) and the Chartered Institution of Building Services Engineers (CIBSE) (CIBSE, 2023) have identified cut points for indoor temperature based on thermal acceptability for most occupants, under simulated environmental and personal factor conditions. These cut points are used to inform thermal conditions in office settings (Wang and Hong, 2020). However, there is a scarcity of temperature cut points specifically tailored for residential settings, or for health outcomes outside of thermal comfort.

The objective of this review was to identify peer-reviewed studies of associations of warm indoor temperatures with mortality/morbidity outcomes or thermal comfort. We summarized the observational population health literature that has estimated associations between indoor warm temperatures with health outcomes. We focused on methods utilized to assess indoor temperature exposures, discussed the advantages and disadvantages of these various approaches and considered potential uncertainties and their implications for understanding associations between indoor temperature and health. When available, we also extracted and reported empirically identified maximum indoor temperature thresholds.

## 2. Methodology

We utilized the Preferred Reporting Items for Systematic Reviews and Meta-analysis (PRISMA) framework (Page et al., 2021) to prepare a protocol for this review. On February 9, 2023, we conducted a search of the following four databases: Web of Science, Global Index Medicus, Ovid Medline, and Embase. Thus, any article that was published and indexed in those databases, as of that date, was eligible for inclusion. Search terms included variations of combinations of words capturing ‘indoor temperature’, ‘morbidity’, or ‘mortality’ outcomes, and ‘thermal comfort’. The specific search terms we used in the corresponding search databases are given in Supplemental Table 1. The initial search generated 1807 total papers from the following search engines: Web of Science (*n* = 654), Global Index Medicus (*n* = 82), Ovid Medline (*n* = 1069), and Embase (*n* = 2). We used Covidence, a web-based tool that allows collaboration between multiple reviewers, to facilitate the article screening and extraction stages of this review (COVIDENCE, 2023).

### 2.1. Selection process and inclusion criteria

The abstract/title and full article review process was conducted by five reviewers (JE, LS, AD, WH, EL). Each abstract and article was reviewed in duplicate, meaning that each article was screened by two authors at each stage. Results were compared and, when necessary, discrepancies were discussed and resolved. Following the abstract review stage, full versions of the article texts were reviewed in duplicate to determine if they met the inclusion criteria. Studies were included if the following criteria were satisfied:

1. written or translated in the English language;
2. included human subjects;
3. warm or hot residential indoor temperatures (either simulated, modeled, or empirically measured) served as a primary explanatory variable;
4. estimated associations with an empirically assessed health, well-being, or thermal comfort outcomes;
5. was an observational study.

We excluded papers for the following reasons: there was no health/well-being/thermal comfort outcome; the outcome was simulated; associations were estimated with cold temperatures, exclusively; the study used an experimental design (e.g., laboratory or controlled hospital settings); the study was set in a non-residential setting (e.g., occupational or school settings, aside from residential dormitories); or the study estimated associations with outdoor temperatures, exclusively. Because we were interested in understanding associations across different levels of daily temperature, rather than across consecutive days of extreme heat, and because a primary objective of this review was to identify empirically estimated temperature thresholds, we also excluded studies that estimated associations with heatwaves coded as a categorical variable (i.e., heatwave day versus non-heatwave day), without any comparison of outcomes between different levels of indoor temperature.

### 2.2. Data collection

Three authors completed the data extraction stage (JE, LS, and AD), during which the following information was extracted from the papers: bibliographical information (author, year, title), study location (country, city, region), study time period (years, seasons, months), overall objective, primary health or well-being outcome, methods used to ascertain the human health or well-being outcome, population demographics (total number of participants, gender, age, race/ethnicity), indoor temperature metric and the temperature exposure assessment methods used, study design, analytic approach including covariates evaluated, and results (effect estimates for associations, including beta coefficients, odds ratios, risk ratios, etc.). Data extraction was conducted in duplicate.

### 2.3. Identifying temperatures thresholds

We collected data on indoor temperature thresholds that were derived empirically. We defined empirically ascertained thresholds as those that were identifed analytically as the temperature after which detriments in health and wellbeing outcomes of interest were observed to occur.

### 2.4. Identifying a priori temperature cut points

Several papers estimated associations with temperature cut-points defined *a priori*. These cut points were based on one of the following: (1) recommended international guidelines (e.g. ASHRAE), or (2) previously published peer reviewed studies. We extracted these cut points, as well. However, because they were not defined empirically, but rather based on *a priori* decisions, we describe them as “*a priori* cut points,” rather than thresholds.

## 3. Results

### 3.1. Papers identified and included

**Fig. 1.**
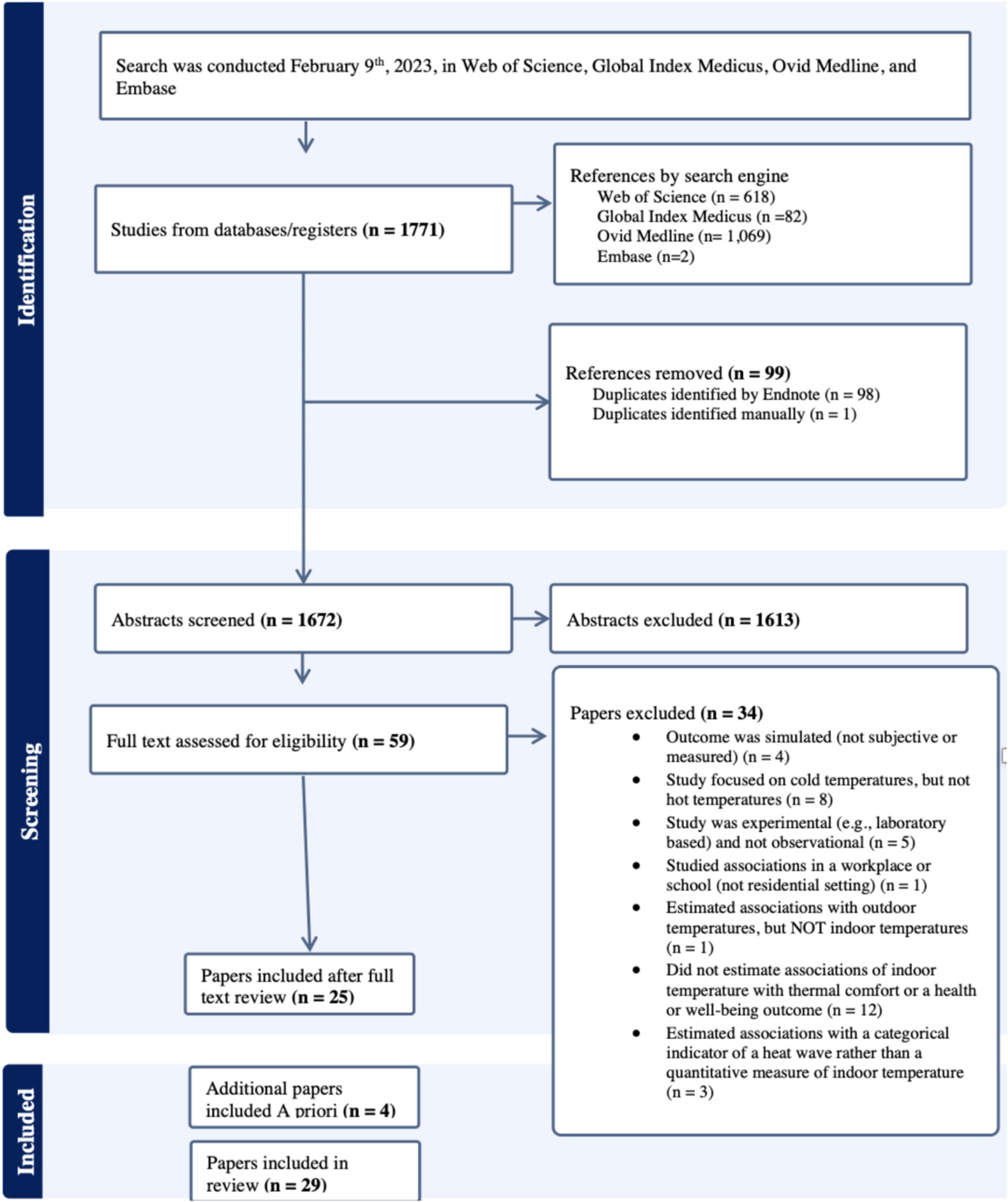
Flow chart showing papers included and excluded from the systematic review.

The search, conducted on February 9th, 2023, yielded 1771 studies through the four search engines (Fig. 1). After duplicates were removed, 1672 remained. After reviewing the title and abstracts of 1672 papers, 1613 were excluded. We reviewed the full text of the remaining 59 articles. Of these, we excluded 34 because the study: simulated the health outcome (*n* = 4), focused on cold temperatures only (*n* = 8), was experimental (*n* = 5), estimated associations outside of residential settings (*n* = 1), estimated associations with outdoor but not indoor temperatures (n = 1), did not estimate associations with a health outcome (*n* = 12), and estimated associations with heatwaves, defined categorically (*n* = 4). We identified three additional papers that our search did not identify through review of reference lists, or expert knowledge. In total, 29 papers were retained for full review.

### 3.2. Study locations and associated climate zones

**Fig. 2.**
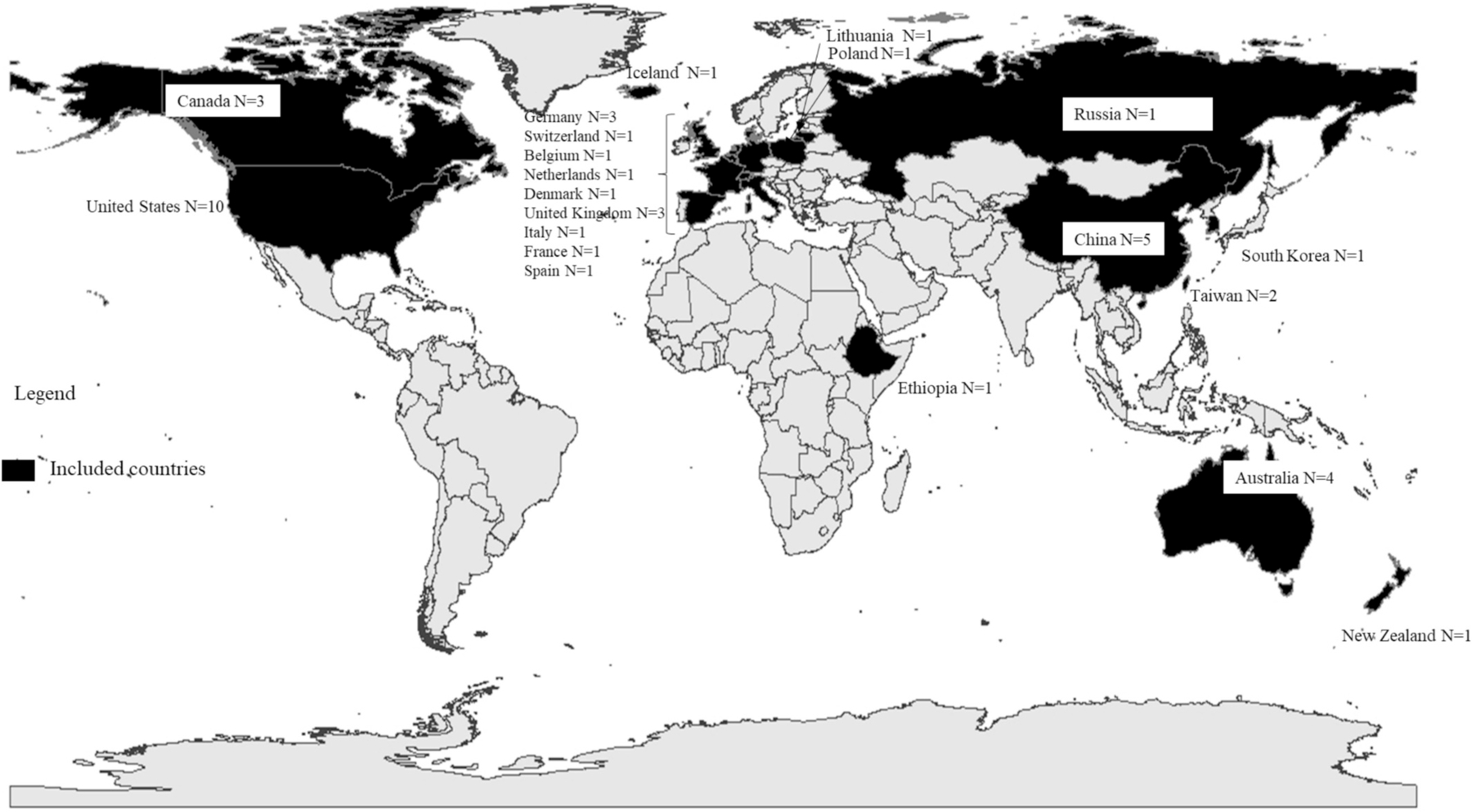
Countries represented by the included studies, and the numbers of studies conducted within each of these locations.

Fig. 2 illustrates the countries represented across the studies. Table 1 provides detailed information on location (city and country), time-period (season), and the objective of the 29 papers included. The earliest publication year was 2007. All continents across the world, except for South America and Antarctica, were represented. North American studies were conducted in Phoenix, Arizona (Ahrentzen et al., 2016); Boston, Massachusetts (Cedeño Laurent et al., 2018); Cambridge, Massachusetts (Williams et al., 2019), Detroit, Michigan (Gronlund et al., 2022); Baltimore, Maryland (McCormack et al., 2016); Houston, Texas (O’Lenick et al., 2020); New York City, New York (Quinn et al., 2017, Uejio et al., 2016); Atlanta, Georgia (Uejio et al., 2022); Montreal and Quebec, Canada (Goldberg et al., 2015); and Quebec, Canada (Teyton et al., 2022). European studies were conducted in Augsburg, Germany (Beckmann et al., 2021); Struttgart, Germany (Lindemann et al., 2017); England, United Kingdom (Sutton-Klein et al., 2021; Vellei et al., 2017); and Arnhem and Groningen, Netherlands (van Loenhout et al., 2016). Asian studies were conducted in Hong Kong, China (Han et al., 2020); Beijing, China (Zhang et al., 2018); various locations in Taiwan (Jung et al., 2020; Jung et al., 2021); Seoul, Korea (Kim et al., 2012); various locations in China (Li et al., 2018), including Xi’an, China (Wei et al., 2022); and Bayannur City, China (Yang et al., 2022); Australian studies were conducted in the Illawarra region, New South Wales (Tartarini et al., 2017) and in the Southern (Hansen et al., 2022) and Northern (Loughan et al., 2015) regions. Lastly, the one African study was conducted in Jimma Town, Ethiopia (Yadeta et al., 2022).

### 3.3. Study populations and common health outcomes

Table 2 shows the study population and health and mortality outcomes studied. Study populations ranged from small, where there were fewer than 100 participants to large with over 100,000 participants. There were also some midsize studies, with participant numbers ranging from 100 to 800. Many of the studies (40.7 %) estimated associations in populations older than 60 years.

Outcomes studied can be broken down into the following three categories: physical health (e.g., ‘cardiovascular disease’), mental health and well-being (e.g., self-reported emotional wellness’), and intermediates, defined as outcomes that are on the casual pathway between indoor heat and acute or chronic health outcomes (e.g., trouble sleeping). A majority of the studies estimated associations with physical health outcomes, including the following: general health physical performance, headaches, cramps, oxygen saturation, acute respiratory illness, non-infectious respiratory diseases, lung function, mean hourly hear rate, mean hourly galvanic skin response, distress medical calls for respiratory cases, breathing discomfort, shortness of breath, pulse rate, cardiovascular disease-related emergency department visits, distress medical calls related to cardiovascular cases, circulatory mortality, circulatory hospitalizations, blood pressure, and distress calls related to diabetes. The mental health outcomes studied included emotional distress, anxiety, and depressive symptoms. Lastly, the intermediate outcomes included sleep quality, cognitive function, agitation related dementia, fatigue, subjective heat stress, objective heat stress, thermal comfort, thermal sensation, annoyance by heat at night, and dry mouth.

### 3.4. Methods used to assess indoor temperature exposures

Indoor temperature exposure assessment methods included use of data sensors, thermometers, data-driven models, or physics-based models (Table 2). In three papers, exposure assessment methods were not reported (Barnett et al., 2007; McCormack et al., 2016; Zhang et al., 2018).

#### 3.4.1. Sensors

The most commonly used exposure assessment method was calibrated sensors (*n* = 19). The following temperature sensors were used: HOBO sensors, Elitech RC-5, CCS811 sensor, DS 18B20, TR-72 U, Dwyer 485, HL-1D, ROTRONIC, DS1923 Hygrochron iButton, and TH22R-EX. The frequency with which the temperatures were recorded by sensors varied. The highest frequencies with which indoor temperature were measured were five-, 10-, and 15-min intervals. These high frequency measurements were collected over the course of the summer months in one-year or two-year study periods. Others collected indoor temperatures hourly during the summer months in one- or two-year periods, daily during a three-year period, and every 4 weeks in the summer months of one year. Five papers did not report the frequency with which indoor temperatures were recorded. In two studies, sensors were used to collect temperature measures at a single point in time. Specifically, in a study of associations between indoor heat and paramedic emergency calls for cardiovascular and respiratory events, temperature measurements were collected in the 4 min after the paramedic’s arrival on the scene and 4 min prior to the paramedic’s departure (Uejio et al., 2016). During these times, measures were recorded at two-minute intervals and then averaged together. Similarly, in a study of associations between indoor temperatures and seasonal thermal comfort, indoor temperatures were measured simultaneously, at two-minute intervals, during a survey interview (Wei et al., 2022).

The locations at which authors placed the data sensors within residences also varied across studies. Most put sensors in one location, such as a bedroom, living room, or a room specified by the participant as most frequently used. The data sensors were mounted at eye level on a wall unexposed to sun or air conditioning units. In a handful of studies, investigators measured indoor temperatures in multiple locations within the participant’s residence; the goal of using multiple sensors was to account for spatial variability within a large residence. Yang et al. (2022) measured indoor temperatures in the master bedroom and living rooms, placing data sensors at 1.1 m above the ground and away from the doors and windows to eliminate potential outdoor temperature draft. However, if the selected rooms measured between 20 and 50 m^2^, measurements were taken at two positions. The investigators then assigned the final exposure as the average of the measures collected at the two spatial points.

In other studies in which indoor temperatures were measured in multiple locations, investigators did not account for spatial variability, nor did they specify where the sensors were placed. For example, Vellei et al. (2017) stated that they measured indoor temperature in both the living room and kitchen of participants’ households but did not specify the setup of the sensors. These authors, however, implemented a multilevel post-processing of temperature data. This process involved the following: (1) filtering and smoothing indoor temperature data (presented as a time series) to eliminate outliers and errors influenced by nearby appliances; (2) visually inspecting the time series by comparing hourly indoor temperature with hourly metrics for occupant radiator temperatures, outside temperature, solar irradiation, and CO_2_ concentration to determine if measurements were affected by outside solar radiation, heating sources, or sensor misplacement; and (3) excluding data from sensors that reported <80 % of the time during the study period.

Some authors provided details about the placement of data sensors. For example, Quinn and Shamman et al. (2017) installed at minimum one sensor at approximately 1.5 m above the ground, on either walls or furniture within the living room. If the home was large (exact measurements were not specified), a second sensor was placed in a bedroom. In addition, van Loenhout et al. (2016) placed data sensors in the living room “at living height” and in bedrooms “at sleeping height” away from any heat and ventilation sources. Lastly, six studies did not indicate the rooms in which the sensors were placed, although in two papers the authors said that they installed monitors away from heat sources (e.g., computer screen, direct solar radiation, etc.) and sources of draft (e.g., air conditioners) (Cedeño Laurent et al., 2018; Williams et al., 2019). In addition, of the six articles that did not specify the rooms in which temperatures were collected, two said that the sensors were positioned 1.1 m above the floor and in rooms in which the residents spent a large portion of their time (Li et al., 2018; Wei et al., 2022) and one (Tartarini et al., 2017) installed sensors at 0.6 m or 1.1 m above the floor to ascertain temperature exposure at chest height and standing height of occupants respectively, where the specific height of the device in each room was selected based on “the most common type of activity and body position of the participant” reported using in the respective room.

Gronlund et al., 2022 used HOBO data sensors to record indoor temperature and humidity levels, and used these measures to calculate the apparent temperature (AT). Lastly, Vellei et al. (2017) used data sensors to describe the difference (dT) between the mean temperature in the occupant room and the ‘comfort temperature’.

#### 3.4.2. Thermometers

In three papers, investigators utilized handheld thermometers to measure indoor temperature. Yadeta et al., 2022 measured temperature using the handheld “AcuRite” digital thermometer. The team held the thermometer at a height of 1.1 m above the floor of the living room of each residential building. Measures were taken six times per day at two hour intervals between 8:00 a.m. and 6:00 p.m. Kim et al. (2012) used an electronic hygrothermograph, which is a chart recorder that measures and records both temperature and humidity, to measure indoor temperatures at 15-min intervals during the morning and afternoon, over a nine day period in two different households. Lastly, Sutton-Kline et al. (2021) collected exposure data using a digital thermometer with a probe, placed on a surface in the participant’s household away from a radiator and out of direct sunlight.

#### 3.4.3. Data driven and/or physics-based modeling (mathematical equations and/or EnergyPlus simulations)

In three papers, investigators estimated indoor temperatures using predictive models or physics-based simulation methods. Jung et al. (2020) and Jung et al. (2021) used a HOBO sensor to collect hourly measures of indoor temperature over the course of the study in a selected number of houses in various locations in Taiwan. The authors then built models to predict indoor temperatures using the following explanatory variables: outdoor meteorological variables (outdoor temperature, relative humidity, atmospheric pressure, wind speed and direction), land surface temperature (from MOD11A2 from National Aeronautics and Space Administration [NASA], US), the normalized difference vegetation index (NDVI) measure of greenness density (MOD13Q1 from National Aeronautics and Space Administration [NASA], US), building characteristic data (average building age, building structure proportion, floor, and area), and occupant behaviors (smoking, cooking, frequency of opening or closing window/front/back door, burning incense, frequency of cleaning floors, use of air conditioners or fans, and electricity consumption). Data on building characteristics and occupant behaviors were ascertained using surveys. The calculation yielded hourly measures of indoor temperatures that were then used to calculate cumulative degree hours for the study period (May to October).

O’Lenick et al. (2020) simulated indoor temperatures in Houston, Texas using EnergyPlus, which is a validated, whole-building energy simulation program developed by the U.S. Department of Energy. EnergyPlus uses physics-based equations to calculate thermal loads in different climate zones and the inputs for the simulation are typically outdoor conditions, occupant behavior, and heat and mass transfer between indoors and outdoors (EnergyPlus, 2023). EnergyPlus models were parameterized based on half- hourly measures of both indoor and outdoor environmental conditions such as temperature, humidity, and carbon dioxide which were gathered at the home of participants during one year of summer months of the total 15-year study period. The authors validated the EnergyPlus for residential buildings by comparing the model’s output with measured indoor parameters of four homes in the study location, and found that the simulated models predicted indoor temperatures with a root-mean square deviation (RMSE) of 0.4 °C,0.4 °C,0.5 °C, and 0.6 °C in the select homes.

#### 3.4.4. Number of participants included in the analysis of temperature-outcomes associations by assessment method

**Fig. 3.**
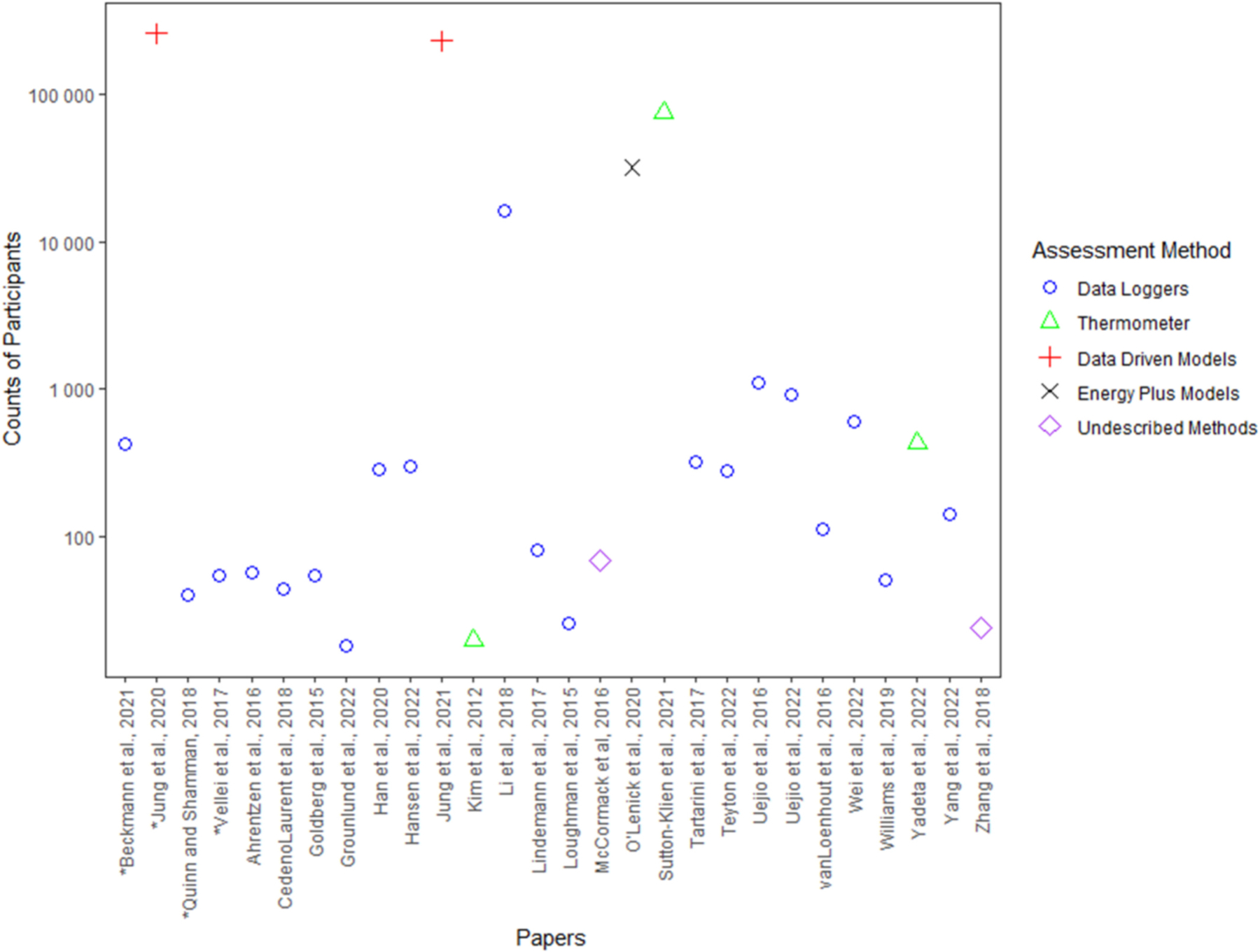
Counts of participants (represented on the log scale) by indoor temperature assessment method. * indicates that the count is based on the number of households because the number of participants was not disclosed. Barnet et al. (2007) was not included because it was a pooled study.

Fig. 3 shows the counts of participants (represented on the log scale) by different indoor temperature assessment methods used across the papers. Studies that used data driven models had a sample of participants that ranged 30 from 260,465, studies using thermometers had a sample of participants that ranged from 20 to 74,736, studies using EnergyPlus models had a sample of participants of 32,043, and studies that used data sensors had a sample of participants that ranged from 18 to 16,458.

### 3.5. Study design, statistical methods and parameterization of indoor temperature

Details on statistical methods and covariates can be found in Table 2. Thirteen studies used a panel or repeated measures design, ten studies were cross-sectional, four used a time stratified case crossover design, and two were case control studies. Statistical analysis methods varied, and included logistic, linear, generalized estimating equations, and Poisson regression models. Not all analyses adjusted for covariates. Among those that did, covariate adjustment sets varied according to the study design and research question. Common covariates included demographic characteristics (e.g., age, sex or gender); environmental conditions (e.g., outdoor air pollutant concentrations such as fine particulate matter or ozone, or precipitation); and behavioral factors (e.g., hydration, caffeine intake, smoking). Other studies accounted for housing characteristics (e.g., floor level of the apartment) and health conditions (e.g., presence of underlying chronic conditions) or temporal factors (e.g., day of the week, major holidays).

Authors parameterized indoor temperature within their analysis in a variety of ways. The majority of studies (*n* = 18) parameterized indoor temperature as a continuous variable. Some, but not all papers accounted for non-linear associations between indoor temperature and outcome variables. For example, Gronlund et al. (2022) modeled indoor temperature using a piecewise linear spline with one inflection point (knot) at the median AT of 22 °C in the exposure dimension and a natural cubic spline with one knot. Jung et al. (2020) and Jung et al. (2021) measured indoor temperatures continuously but only estimated associations with the cumulative hours spent in temperatures that ranged from 27 °C to 31 °C.

The remaining studies parameterized indoor temperatures as either a binary (*N* = 4) or categorical (N = 4) variable. Specifically, for authors that used binary cutoffs, cut points were as follows: 27.2 °C (Ahrentzen et al., 2016), 24.9 °C (Beckmann et al., 2021), 28 °C (Hansen et al., 2022) and 18 °C (Sutton-Klein et al., 2021).

Four studies parameterized indoor temperatures categorically. Lindmann et al. (2017) utilized multilevel linear regression models in a repeated measures panel study design and categorized indoor temperature into 5 bins (<22 °C, 22–23.9 °C, 24–25.9 °C, 26–27.9 °C, >27.9 °C). Teyton et al. (2022) utilized generalized estimating equations and a cohort study design and categorized indoor temperature in terciles (T2 (28–30 °C) and T3 (30–33 °C) relative to T1 (18–22 °C). Similarly, Cedeño Laurent et al. (2018) utilized a repeated measures design with generalized estimated equations and categorized indoor temperature based on quartiles; however, the exact distribution was not specified. Lastly, Uejio et al. (2016) categorized indoor temperature corresponding to the ~73rd, 83rd, and 88th percentiles of the distribution for heat index (≥25, 26, and 27 °C respectively).

### 3.6. Summary of findings across included papers

**Fig. 4.**
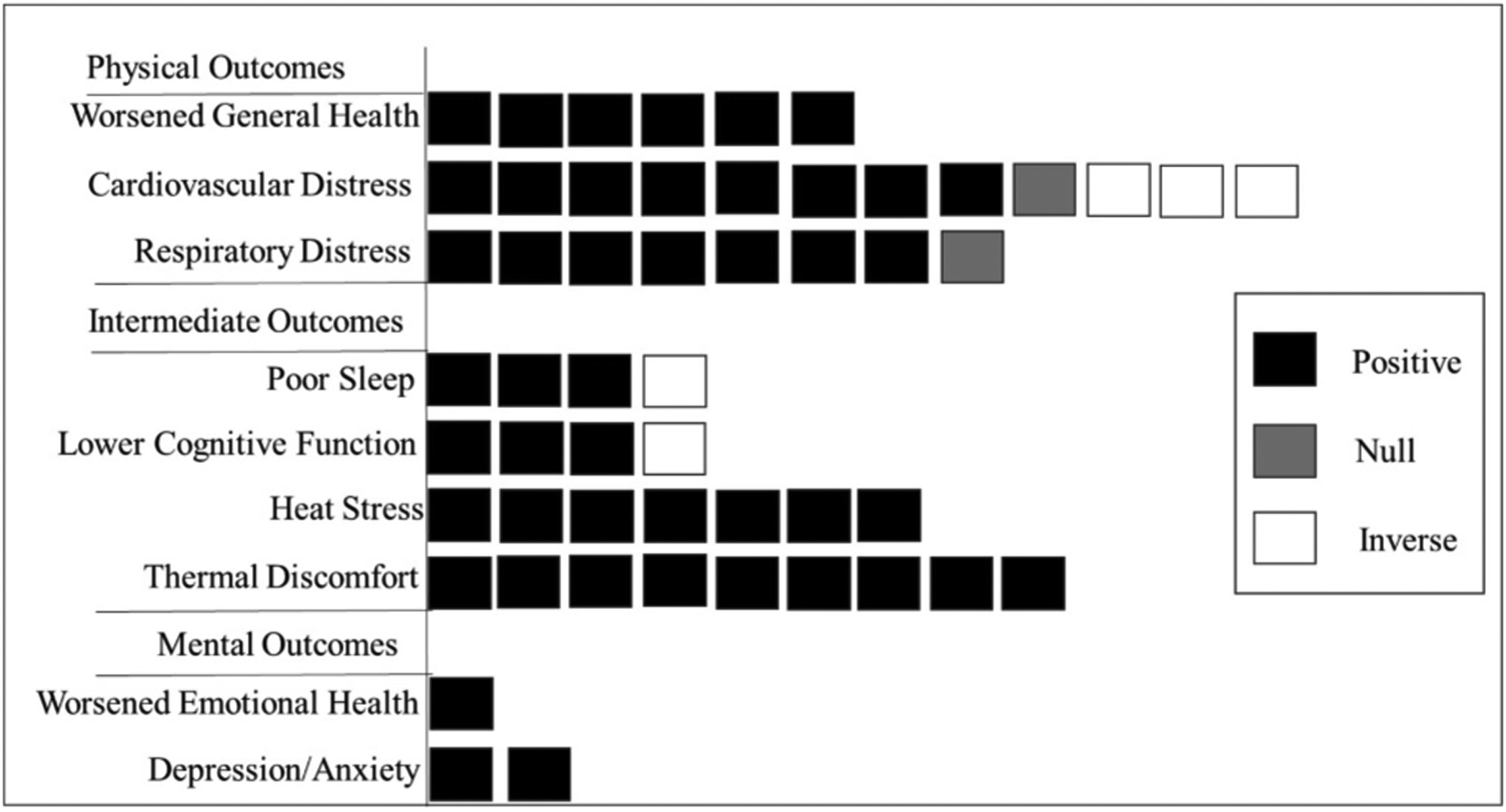
Main findings from studies that estimated associations of indoor temperature and health outcomes. The black box indicates estimates (beta coefficients/ OR/ RR) of association that are >0 (beta coefficients) or 1 (OR/RR) denoted as a positive association, the white box indicates estimates (beta coefficients/ OR/ RR) of association that are <0 (beta coefficients) or 1 (OR/ RR) denoted as an inverse association, and the gray box indicates estimates (beta coefficients/ OR/ RR) of association that are equal to 0 (beta coefficients) or 1 (OR/ RR) denoted as a null association.

Fig. 4 shows the main findings by category of outcome (physical outcomes, intermediate outcomes, and mental health outcomes). Physical outcomes were categorized into three subcategories (worsened general health, cardiovascular distress, and respiratory distress), intermediate outcomes were categorized into 4 subcategories (poor sleep, lower cognitive function, heat stress, and thermal discomfort), while mental health outcomes were categorized into 2 subcategories (emotional health and depression/anxiety). Supplemental Table 2 shows the health outcomes in each subcategory.

Overall, results from across the papers suggest that warmer indoor temperatures are associated with decrements in a variety of physical health, mental health, and intermediate outcomes that facilitate well-being– across different study populations and settings. In most studies, warmer indoor temperatures were linked with poor outcomes, including reduced cognitive function, perceived heat stress, shortness of breath, thermal discomfort, respiratory illness. A handful of papers found that systolic blood pressure decreased in association with warmer indoor temperatures (Barnett et al., 2007; Goldberg et al., 2015; Kim et al., 2012). However, contrary to a priori hypotheses, Gronlund et al. found that warmer indoor apparent temperatures were associated with improved, rather than reduced, cognitive performance, and that warmer nighttime temperatures were associated with less daytime sleepiness (2022). While Uejio et al. reported higher odds of distress calls for respiratory outcomes in association with warm indoor temperatures, associations with distress calls for cardiovascular outcomes were null (2016).

Some, but not all papers, explored heterogeneity of associations between indoor temperature and outcomes. The following factors were explored as modifiers: sex (Jung et al., 2020; Jung et al., 2021), age (Jung et al., 2020), urban/rural location (Li et al., 2018), initial physical ability (gait speed) (Lindemann et al., 2017), and untreated versus treated hypertension (Kim et al., 2012). Others evaluated differences according to categories of outdoor temperature (Sutton-Klein et al., 2021) season (Wei et al., 2022), or air pollution levels (McCormack et al., 2016). One paper evaluated effect modification by the following census block level compositional measures: racial composition, proportion living below the poverty line, and proportion living alone (O’Lenick et al., 2020).

For example, Jung et al. (2020) found that associations between indoor temperature and emergency department visits for cardiovascular disease were more substantial among men than women, and for those aged 85 and older versus younger individuals. Jung et al. (2021) found that associations of warmer indoor temperatures (measured as the cumulative number of hours during the cooling season that were higher than a threshold temperature) were associated with modestly higher risk of emergency department visits for infectious and non-infectious respiratory diseases in women than in men. More details on the results of each of the included studies can be found in Supplemental Table 3.

#### 3.6.1. A priori cut points

In four papers, investigators used a temperature cut point that was specified, *a priori*, based on established standards (Table 3). Ahrentzen et al. (2016) utilized the American Society of Heating, Refrigeration and Air Conditioning Engineers (ASHRAE) standards (ASHRAE, 2023). Specifically, the authors measured how sleep, emotional health, general health, and thermal comfort were affected when indoor temperatures exceeded the ASHRAE standard of 27.2 °C in older people (ages 62–92, *n* = 57) living in Arizonia. These authors found that when following up on a cohort, individuals that lived in temperatures that were maintained below the a priori threshold corresponded with improvements in occupants’ reported sleep, emotional health, and general health but not for their perception of thermal comfort. Another study utilized a cut point established by the Chartered Institution of Building Services Engineers (CIBSE) (CIBSE, 2023). Specifically, Beckmann et al. (2021) measured how subjective heat stress during sleep of individuals of all ages (*n* = 427) living in Germany was affected when temperatures within participant’s bedrooms exceeded 24 °C and found significant difference in increased subjective heat stress among individuals living in temperatures above the *a priori* threshold. Tartarini et al. (2017) used cut points based on International Organization for Standardization to measure associations between dementia related agitation and dementia related disruptiveness at temperatures outside a range of 20 °C to 26 °C for 325 older participants in New South Wales, Australia, and found that cumulative exposure to temperatures warmer than 26 °C were linearly correlated with dementia related health outcomes (Tartarini et al., 2017). Lastly, one study did not rely on established guidelines but implemented cut points based on previous peer reviewed literature. Specifically, Gronlund et al. in 2021 utilized a threshold of 22 °C because the authors hypothesized that their outcomes (cognitive function and sleepiness) would increase with increasing temperatures above 22 °C, based on prior literature. In line with their hypothesis these researchers found that sleepiness scores decreased when nighttime indoor temperatures conditions remained below 22 °C.

#### 3.6.2. Empirically quantified temperature thresholds

Fourteen papers empirically quantified a temperature threshold, which we define as the temperature after which risk or rates of the adverse health outcome of interest began to change (i.e., increase or decrease depending on the outcome). Of the fourteen papers, seven estimated thresholds for subjective thermal comfort (thermal sensation vote, thermal comfort, thermal acceptance, and thermal preference). In four papers, investigators identified thresholds for thermal sensation which is a subjective evaluation and represents sensation of thermal comfort in a given environment. First, Wei et al. (2022) studied adults of all ages (*n* = 526–609) in different Chinese cities. The authors observed declines in thermal sensation vote after indoor temperatures reached 17.9 °C in the spring and 26.1 °C in the summer. Yadeta et al. (2022) studied adults of all ages (*n* = 430) in Ethiopia. The authors observed declines in thermal sensation after indoor temperatures reached 20.4 °C. Yang et al. (2022) studied adults of all ages (*n* = 141) in China and observed declines in thermal comfort after indoor temperatures reached 24.8 °C in the city, 20.4 °C in towns, and 18.2 °C in rural areas. Lastly, Zhang et al. (2018) studied university students (*n* = 24) in Bejing China and observed that acceptable temperature ranges for thermal sensation vote was between 22.2 and 28.8 °C while students were asleep and between 21.1 and 27.3 °C while students remained awake in their dorm rooms.

Two papers estimated thresholds after which thermal comfort, which is a subjective assessment of how comfortable people feel with the temperature, declined. First, Hansen et al. (2022) studied older adults, ages 61–98 years old (*n* = 303) in Australia. They observed declines in thermal comfort after indoor temperatures reached 28 °C. Loughnan et al. (2015) studied adults ages 55 years and older (*n* = 26) in Australia and observed declines in thermal comfort after temperatures reached 26.6 °C.

Thermal acceptance is a subjective assessment of whether the temperatures are within a range that one finds acceptable and conducive to well-being and activities. Wei et al. (2022) found that the acceptable temperature range for 90 % of their study population (adults in different Chinese cities) was between 19.2 °C and 27.0 °C. These same authors also observed thermal preference, which reflects the degrees of satisfaction concerning the current thermal environment and found that the preference declined with temperatures warmer than 23.2 °C and 25.6 °C for spring and summer respectively.

Seven other papers quantified maximum thresholds for the following outcomes: sleep and cognition, agitation, health/welling, physical performance, distress calls for respiratory illness, diabetes, and cardiovascular calls, mean hourly heart rate, mean hourly galvanic skin response to heat, and various intermediate health outcomes including trouble sleeping, urination frequency, thirst, and dry mouth. Specifically, Cedeño aurent et al. (2018) identified 22 °C as the threshold after which reports of poor sleep and reduced cognitive function began to increase, in individuals aged 18 to 29 years (*n* = 44) in Boston, MA, USA. Tartarini et al., in 2017 identified 22.6 °C as a temperature that would increase agitation in an older population aged >60 years and who suffered from dementia, in New South Wales Australia. Additionally, Uejio et al. (2022) identified 21.1 °C and 24.6 °C as the temperatures after which the odds of emergency calls for diabetes and respiratory distress were higher than controls, respectively, among adults living in Atlanta, Georgia (median age:57). Uejio et al. (2016) identified 26 °C as the temperatures after which the odds of emergency calls for cardiovascular distress were higher than controls among adults living in New York city (median age:52).

Lindemann et al. (2017) identified 27.9 °C as the indoor temperature after which there was evidence of decreased physical performance in adults aged 60 years and older (*n* = 81) in Stuttgart, Germany. Hansen et al., in 2022 studied older adults, ages 61–98 years old (*n* = 303) in Australia, and observed declines in perceived health with temperatures above 24.3 °C. Additionally, William et al. (2019) reported findings indicating that increasingly warm indoor temperatures could affect physiological markers in a small sample of older adults (*n* = 51, mean age 65 years) living in Cambridge, Massachusetts. Specifically, temperatures above 24 °C were associated with changes in mean hourly heart rate and galvanic skin response (Williams et al., 2019). Lastly, Teyton et al., in 2022 studied an older population, aged >60 years, in Canada and observed temperature thresholds in several poor health outcomes, such as trouble sleeping, less urination, thirst, and dry mouth at 20 °C, 22 °C, 18 °C, and 24 °C respectively.

## 4. Discussion

Given that most people spend the majority of their time indoors and at home, there is a critical need to improve the understanding of links between indoor temperatures and health (Klepeis et al., 2001; Centers for Disease, 1994). In this review, we identified and summarized peer reviewed literature that quantified associations of indoor temperatures with physical health, mental health, and intermediate outcomes. Thermal discomfort, worsened general health, and respiratory distress were among the most studied health or well-being outcomes. We found that warmer indoor temperatures were often, though not always, associated with poor outcomes. Although our search identified papers describing results from studies in most continents, South America and Antarctica were not represented. Meanwhile, North American studies were over-represented, with 10 studies based in the United States and 3 conducted in Canada.

We identified four distinct approaches for estimating indoor temperature exposures: (1) longitudinal or cross-sectional measurement using data sensors inside the home, (2) capturing temperature with a one-time thermometer measurement, (3) developing data driven models, and (4) using physics-based simulations to predict indoor temperatures. We also found that fourteen papers empirically quantified indoor heat exposure thresholds based on observed health effects in their study populations. These thresholds varied, from 18 °C to 35 °C. The thresholds were calculated in different geographic areas, in different study populations, and for different outcomes. Taken together, this small body of evidence suggests that one size does not fit all when it comes to identifying an optimal threshold temperature. In the proceeding sections, we will discuss the advantages and disadvantages of these methodologies and provide recommendations for future research. In addition, in the last section we will discuss how temperature cut points were created or identified, as well as the related public health implications of these thresholds.

### 4.1. Indoor temperature exposure assessment methods

The majority of studies utilized either data sensors or digital thermometers to empirically measure indoor temperatures. Each of these methods has its own set of advantages and drawbacks. Data sensors allow for remote, continuous, and consistent measurement over extended periods, which can be particularly important for investigators to assess short-duration extremes or long-term trends. Despite the advantages of data sensors, they may still be subject to some measurement error. For example, the way in which data sensors are installed may impact the results. Some researchers included in this review emphasized the importance of keeping the data sensors away from heat sources that may highly influence results, such as the sun or other heating/cooling devices (Cedeño Laurent et al., 2018). In addition, researchers must weigh the costs and benefits of purchasing more than one device per household in longitudinal analyses, as multiple devices allow investigators to account for spatial variability within large indoor environments and occupant movement patterns (Yang et al., 2022).

While individually, these sensors do not cost a lot, with prices ranging from $260 USD to $300 USD in 2024, costs can escalate rapidly when purchasing multiple units, especially if researchers aim to estimate associations across a large population or wish to acquire sensors for each room in a participant’s residence. Lastly, data sensors are more expensive than thermometers.

Thermometers are a simple and portable way to immediately assess temperatures in an indoor environment. These may be particularly beneficial in cross-sectional studies. However, the use of thermometers can have many disadvantages. To name a few, thermometers are subject to human error due to incorrect reading and interpretation, and often do not possess the ability to store or record data over time. With both approaches – thermometers and data sensors – sample sizes may be limited when compared with data-driven or simulation approaches. There are benefits (both financially and with respect to time) associated with using data driven or physics-based simulations, as they do not require primary data collection. The use of these approaches may also facilitate inclusion of a larger number of households and use of existing data sources. By allowing investigators to estimate exposures for a larger group of households, these approaches may also facilitate conducting studies across various geographic areas or climate zones, which enables contrasting results across the different areas. However, energy simulations or data driven models may be limited due to their computational intensity, requirements for specific data inputs, and susceptibility to uncertainties. These uncertainties include model assumptions that may not accurately reflect real-world conditions, such as occupant behavior, as well as inaccuracies in meteorological data.

### 4.2. Indoor temperature thresholds

Identifying safe indoor temperature thresholds is important for informing thermostat setting recommendations, safe building standards, and the creation of policies intended to protect the health of tenants and housing occupants. Currently, only a handful of locations have created policies that regulate upper indoor temperatures. In Toronto, Canada, the Property Standards Bylaw requires landlords to keep air conditioners running between June 2 and September 13, and to maintain an indoor temperature of no warmer than 26 °C (Merali, 2023; Toronto Municipal Code Chapter 497, 2018). Montgomery County, Maryland USA recently ordered landlords of apartment buildings to “supply and maintain” air conditioning units to ensure that indoor environments remain at 80 degrees °F (26.7 °C) or less during the summer months (Montgomery County, Maryland: Bill 24–19, 2020). However, this mandate covers rental apartments, only, and exempts detached single-family homes (Tan, 2020). Similarly, Tempe Arizona requires that all rental housing units have cooling systems, and that these systems be equipped to maintain a temperature of 88 °F (31.1 °C) or 82 °F (27.8 °C) by evaporative cooling or by air conditioning, respectively (City of Tempe: Thermal environment, 2024). Moreover, Dallas County, Texas has an ordinance requiring landlords to provide air conditioning systems that maintain an indoor temperature of either 85 °F (29.4 °C) or one that is 20 °F (−6.7 °C) lower than the outdoor temperature (whichever is warmer) (City of Dallas. SEC 27.11). Based on the results from this review the established thresholds presented by policy may be too elevated to support health and comfort.

In fourteen of the papers included in this review, investigators empirically identified thresholds with one paper reporting a threshold as low as 18 °C. The authors used observational data to identify quantitative thresholds. This is important, because heat vulnerability may vary according to health outcome, to population characteristics (e.g. age, underlying chronic conditions), occupant behavior (e.g., clothing worn, hydration), building design, and local climate (De Dear and Brager, 2002). For example, Hansen et al. (2022) found different relevant thresholds according to the outcome: 28 °C for poor thermal comfort, but above 24.3 °C for poor perception of health and well-being.

A few studies relied on temperature cut points established by organizations such as ASHRAE (Ahrentzen et al., 2016; Loughnan et al., 2015), CIBSE (Beckmann et al., 2021) or the International Organization for Standardization (ISO) (Tartarini et al., 2017) to define heat exposures. These are designed to protect a wide range of populations, including occupants of residential and commercial buildings, industrial workers, and the public. However, the use of these cut points for categorization of indoor temperatures in observational studies of morbidity/mortality outcomes limits identification of temperature thresholds for health effects. Safe maximum thresholds may vary according to regional climate differences, and social/biologic circumstances. For example, Ahrentzen et al., 2016 utilized ASHRAE temperature cut point of 27.2 °C to define heat exposure in an older population of individuals (ages 62–92) living in Phoenix Arizonia. The group found that decreases in an apartment’s temperature, below 27.2 °C, was associated with improvement in participant’s self-reported health but not thermal comfort. It is possible that the authors missed associations with thermal comfort because the established cut point was too high for an older population, especially those living in a hot desert climate. Also, these thresholds are created by international committees that are made up of professionals including engineers, manufacturers, and industry experts, all of whom may not have a public health focus. These temperatures cut points are in place to provide guidance for the design, construction, and operation of buildings with the objective to advance industry’s practices, promote energy efficiency and technologies and may not be etiologically relevant for all populations or outcomes (ASHRAE, 2023, International Organization for Standardization, 2005). In addition, although established cut points from ASHRAE, CIBSE, or ISO are updated every few years to account for rising temperatures, these guidelines do not account for important individual or regional-level differences, nor are they targeted, specifically, at residential settings.

### 4.3. Additional gaps

Investigators included in this review studied a variety of health outcomes associated with indoor temperature exposures, including intermediate variables, such as impaired sleep or thermal comfort, mental health outcomes, such as cognition, and physical outcomes including acute respiratory and cardiovascular measures. However, only one study investigated associations with mortality outcomes, pointing to an important knowledge gap regarding how extreme temperatures are related to this outcome (O’Lenick, et al., 2020). Future research that identifies indoor temperature thresholds to prevent premature death is critical. We also found that most research, to date, has been conducted in higher income locations, and has been predominantly in the continents of North America, Australia, and Asia. Additional research in more varied locations is needed, particularly since associations between temperature and health outcomes may vary according to region, climate zone, and residential building design.

### 4.4. Strengths and limitations of this review

While research on links between workplace or school indoor temperature exposures with health or well-being outcomes, including cognitive function, acute respiratory or cardiovascular outcomes, has been reviewed previously, there has been little focus on indoor exposures within residential environments (Zhang et al., 2019). This review paper fills this gap. The strength of the review includes a structured search, capturing articles published through 2023 from various search databases and disciplines. Our focus and detailed description of exposure assessment approaches may be used to inform future study design. We also extracted detailed information on associations between indoor temperature and morbidity or mortality outcomes from a wide range of study populations, and countries. However, this review also has limitations. A relatively small number of articles fit our inclusion criteria and an even smaller sample empirically quantified thresholds, which precluded derivation of any pooled quantitative estimates. Lastly, though we aimed to systematically identify peer reviewed literature describing observational studies of links between residential indoor temperatures and health or well-being outcomes, despite our best efforts, we subsequently identified several relevant papers that our initial search terms did not capture. These articles were documented and incorporated into the review. We also acknowledge the possibility that additional relevant studies may have been unintentionally overlooked.

## 5. Conclusion

Drawing from observational studies, this review paper summarized the current state of the evidence pertaining to the relationship between warm residential indoor temperatures and health or well-being outcomes. We found evidence that warmer indoor temperatures are associated with a variety of adverse outcomes, based on a selection of papers in which investigators used a variety of methods to estimate temperature exposures. However, the number of articles that empirically identified maximum safe temperatures (i.e, thresholds) was limited, pointing to a strong need for further research moving forward. Empirically identifying thresholds have policy and environmental regulation implications. For example, establishing indoor temperature thresholds may shift responsibility to building managers or landlords to maintain cool environments and to the government to enforce such regulations. Amidst the escalating temperatures driven by climate change, the imperative to better understand links between indoor environmental conditions and health, and to inform indoor temperature thresholds, will become increasingly critical.

## Supplementary Material

Supplemental file

## Figures and Tables

**Table 1:** Location and observed seasons of included studies.

Continent	Author, year	Location (country, city, region)	Objective	Study dates and season
Multi continent	Barnett et al., 2007	16 countries (Australia, Belgium, Canada, Denmark, France, Germany, Iceland, Italy, Lithuania, New Zealand, Poland, Russia, Spain, Switzerland, UK, USA)	Estimate the association between systolic blood pressure and indoor and outdoor temperature.	1979–1997, All seasons
North America	Ahrentzen et al., 2016	United States, Phoenix, Arizona	Ascertain if energy retrofits correspond with improved health and comfort.	Before retrofits Jul 2009–Jan 2011 During retrofits: Feb 2011–Aug 2011 After retrofits: Sep 2011–Oct 2012 All seasons
	Cedeño Laurent et al., 2018	United States, Greater Boston area, Massachusetts	Examine relationships between indoor environmental conditions and sleep and cognitive function among student dormitory residents.	July 9–July 20, 2016, One summer month.
	Goldberg et al., 2015	Canada, Montreal and Quebec	Estimate associations of air pollution and meteorological variables with daily exacerbations, symptoms, and physiologic indicators among people with heart failure.	June 2008–January 2011 All seasons
	Gronlund et al., 2022	United States, Detroit, MI	Estimate associations of indoor temperatures with cognitive function and daytime sleepiness in low-income residents.	July–November 2019 Summer and Fall
	McCormack et al., 2016	United States, Baltimore, MD area	Estimate associations of indoor and outdoor temperatures with daily respiratory symptoms, rescue medication use and lung function, among participants with COPD.	2009–2011, Warm weather season, defined as the time between the first and last day that the maximal outdoor temperature exceeded 90 °F in Baltimore for each calendar year.
	O’Lenick et al., 2020	United States, Houston, TX	Estimate associations between indoor heat and diseases of the circulatory system and heat related illnesses.	2000–2015, Summer
	Quinn and Shamman, 2018	United States, New York City, NY	Estimate associations of measured and perceived indoor climatologic conditions with health symptoms.	2013–2015, Winter and Summer
	Teyton et al., 2022	Canada, Quebec	Estimate associations between indoor heat and various physical and mental health symptoms.	2017–2018 Summer
	Uejio et al., 2016	United States, New York City, NY	Estimate associations of hotter indoor conditions with cardiovascular or respiratory case events.	2013 Summer
	Uejio et al., 2022	United States, Atlanta	Estimate associations between indoor heat exposure and exacerbations of respiratory illness and diabetes that are reflected through 9–1-1 call incidents and patient care reports.	2016 Summer
	Williams et al., 2019	United States, Cambridge, MA	Estimate associations between indoor temperature during an extreme heat event and personal physiological responses to heat (mean hourly heat rate and mean hourly skin response).	2015 Summer
Europe	Beckmann et al., 2021	Germany, Augsburg	To identify indoor temperature thresholds for subjective heat stress during heatwaves.	2019 Summer
	Lindemann et al., 2017	Germany, Stuttgart in Southern Germany	Estimate associations between indoor temperature and physical performance in older adults.	2015 Spring, Summer, Fall, with a focus on heat waves during the summer
	Sutton-Klein et al., 2021	UK, England	Assess associations between indoor temperature and self-rated health.	2003–2014 (excluded 2004) Spring, Summer, Winter, Fall
	Vellei et al., 2017	UK, England, Exeter	Estimate overheating, ventilation, thermal comfort, and indoor air quality in newly retrofitted social housing occupied by vulnerable and non-vulnerable households.	2014–2015 Spring, Summer
	van Loenhout et al., 2016	Netherlands, Arnhem & Groningen	Estimate associations of indoor temperatures with heat-related health problems among older individuals.	2012 Spring and Summer
Asia	Han et al., 2020	China, Hong Kong Island	Estimate associations of household environmental conditions, including temperature, relative humidity, and absolute humidity, with acute respiratory illness.	2016–2018 Spring, Summer, Winter, Fall
	Jung et al., 2020	Taiwan, 11 different cities	Investigate the cumulative effects of warm indoor temperature exposure on the risk of cardiovascular disease related emergency department visits among older adults.	2001–2014 Spring, Summer, Fall, Winter
	Jung et al., 2021	Taiwan, 317 townships	Estimate the cumulative effects of indoor temperature exposure on emergency department visits for infectious and non-infectious respiratory diseases among older adults; estimate heterogeneity according to gender.	2006–2014 Spring, Summer, Fall
	Kim et al., 2012	Korea, Seoul	Estimate the acute effects of heat stress on body temperature and blood pressure in older adults living in poor housing conditions.	2010 Summer
	Li et al., 2018	China, Nine typical cities across five climate zones	Investigate associations between indoor thermal environments and thermal comfort in residential buildings, and how these associations differ by climate zones.	2008–2011 Spring, Summer, Fall, Winter
	Wei et al., 2022	China, Xiân	Investigate thermal comfort requirements in residential buildings and establish an adaptive thermal comfort model tailored to the cold zone of China.	2012, 2013, 2016, 2017 Spring, Summer, Fall, Winter
	Yang et al., 2022	China, Bayannur City	Investigate similarities or differences in indoor thermal environments and thermal comfort across cities, towns, and rural residences.	2019–2020 Fall, Winter, Early Spring
	Zhang et al., 2018	China, Beijing	To determine the relationship between indoor temperature and sleep quality.	2017 Seasons/months not presented; Study conducted over six-week period, excluding weekends and holidays
Australia	Hansen et al., 2022	Australia, Greater Adelaide metropolitan area; the Iron Triangle; the Fleurieu Peninsula.	Investigate how the indoor thermal environment of housing affects the health and wellbeing of older South Australian adults.	2020 Nine-month period including Summer and Winter
	Loughnan et al., 2015	Australia, “a northern Victorian regional town”	Estimate associations between housing characteristics and heat wave resilience in older adults.	2012 Summer
	Tartarini et al., 2017	Illawarra region, New South Wales	Investigate the association between indoor air temperature and agitated behaviors among people with dementia.	2015 Spring, Summer, Winter, Fall
Africa	Yadeta et al., 2022	Ethiopia, Jimma town	Investigate thermal comfort and adaptive measures in naturally ventilated residential buildings in Ethiopia.	2020 Winter and Spring

**Table 2:** Description of outcomes, study population, temperature metrics used in papers included in this review.

Author, year	Outcome	Study population	Indoor temperature exposure assessment methods	Study design	Analysis	Brief description of results^1^
Ahrentzen et al., 2016	Sleep quality, emotional health, general health/quality of life, and thermal comfort	57 total participants. Men and women, ages ranged from 62 to 92 years.	HOBO U-10–001 sensors, recorded every 15 min.	Panel, or repeated measures longitudinal study	Fixed effects ordinal regression Adjusted for floor level of apartment unit	Decreases in apartment’s temperature corresponded with improvements in participants self-reported health but not improvements in thermal comfort.
Barnett et al., 2007	Systolic blood pressure	115,434 total participants. Men and women, ages 35–64 years.	Not specified in paper	Cross-sectional	Bayesian hierarchical models. Adjusted for season, sex, type of sphygmomanometer.	1 °C increase in indoor temperature was associated with 0.31 mmHg reduction in systolic blood pressure.
Beckmann et al., 2021	Subjective heat stress	427 households, Men and women, all ages, including those under 18 years of age.	Elitech RC-5 sensors recorded indoor dry bulb temperature every 15 min.	Cross-sectional	Paired *t*-test; Mann-Whitney-*U* test, and Linear regression analysis. Adjusted for age younger than 65, local climate zone. Analyses stratified by nonvulnerable groups, people who identified as living alone, people with chronic diseases, and people with higher heat exposure (living on a high floor or living in a hot local climate zone).	Warmer indoor temperatures associated with higher subjective heat stress, especially among some subgroups, including people living alone and people with chronic disease.
Cedeño Laurent et al., 2018	Cognitive function	44 university students, 24 living in air conditioned buildings and 20 in non air conditioned buildings. Men and women, ages 18–29 years.	Netatmo indoor air quality monitor recorded indoor dry bulb temperature continuously.	Panel, or repeated measures longitudinal study	Differences-in-differences with generalized linear mixed-effect models. Adjusted for averages of nighttime noise, absolute humidity, carbon dioxide concentrations, hydration, caffeine intake, and time from waking up. Penalized spline terms used to accommodate nonlinear associations.	Residents in non-AC buildings experienced significant cognitive performance reductions during heat waves.
Goldberg et al., 2015	Oxygen saturation (%), pulse rate, systolic blood pressure (mm Hg), Diastolic blood pressure (mm Hg), Self-rated health, and shortness of breath at night	55 total participants with congestive heart failure. Men and women Ages 35 years and older.	HOBO sensors measured indoor air temperature continuously.	Panel, or repeated measures longitudinal study	Mixed random effects model using restricted maximum likelihood estimation. Adjustment set varied across outcomes. Included month of the year, gender, salt consumption in previous day, being sick and having sleeping problems, age, weight, body temperature, number of cups of liquid.	Diastolic blood pressure, self rated health, and shortness of breath at night increased with increases in temperature; however, systolic blood pressure decreased with increases in temperature.
Gronlund et al., 2022	Cognitive function and sleepiness	18 total participants. Men and women ages ranged from 28 to 77 years.	HOBO sensors recorded indoor temperature and dewpoint hourly at night. Apparent temperature (AT) calculated using the following equation = −2.653 + (0.994 × temperature) + 0.0153 × (dew point)^^^2	Panel, or repeated measures longitudinal study	Linear regression models. No adjustment, no stratification. Nonlinear and lagged associations were modeled using distributed lag nonlinear model functions. Included fixed effects for each participant to adjust for time invariant factors. No adjustment, no stratification.	Warmer indoor apparent temperatures (AT) above 22 °C were associated with improved cognitive performance. Warmer nighttime temperatures (above 22 °C) were associated with reduced daytime sleepiness.
Han et al., 2020	Acute respiratory illness	285total participants. Men and women, ages 65 years and older.	HOBO sensors recorded temperature and relative humidity hourly.	Time stratified case-crossover	Conditional logistic regression models. Adjusted for precipitation, PM_2.5_, and O_3_	Weak negative but imprecise associations between indoor temperatures and odds of acute respiratory illness.
Hansen et al., 2022	Self-reported general health and quality of life, thermal comfort satisfaction	71 total participants in home monitoring study. Men and women, ages 61–98 years.	HOBO sensors recorded indoor dry bulb temperature globe temperature, and relative humidity hourly.	Panel, or repeated measures longitudinal study	Stepwise linear regression. No adjustment, no stratification.	Perceptions of health and well-being decreased when indoor temperatures were warmer than 28 °C.
Jung et al., 2020	Cardiovascular disease-related emergency department visits	260,465 total participants. Men and women ages 65 years and older.	Prediction models and the following variables were used to estimate hourly indoor temperatures: hourly outdoor temperature and relative humidity, land surface temperature, normalized difference vegetation index (NDVI), building characteristics, occupants’ behavior, and electricity consumption data The indoor temperature predictions were used to calculate cumulative degree hours exceeding 27° C in a day.	Time stratified case-crossover	Conditional logistic regression models, adjusted for NOx, PM_2.5_, and O_3_, day of week, holiday, and long term time trends. Modeled cumulative degree hours using distributed lag nonlinear model (DLNM) functions. Estimates were stratified by age and sex.	Cumulative exposure to indoor temperatures above 27 °C associated with higher risk of cardiovascular emergency department visits. In stratified analysis, men tended to have higher relative risks than women. In addition, though who were aged >85 years had higher relative risks.
Jung et al., 2021	Infectious and non-infectious respiratory diseases	231,282 total participants gathered. Men and women; ages 65 years and older.	Prediction models and the following variables were used to estimate hourly indoor temperatures: indoor humidity (%), atmospheric pressure (mmHg), global solar radiation (Million-Joule/m2), wind speed (m/s), and wind direction (o), land surface temperature (°C), NDVI, electricity consumption (kilowatt-hour/day), and building characteristics (building structure type: stone or reinforced concrete, building age (year), building level (floor)) between years 2006 and 2014. The indoor temperature predictions were used to calculate cumulative degree hours exceeding 27°C in a day.	Time stratified case-crossover	Quasi-Poisson regression models Adjusted for NOx, PM_2.5_, and O_3_, day of the week, holiday, and long term time trends. Estimates were stratified by gender. Temperature was modeled using distributed lag nonlinear model functions.	Cumulative exposure to indoor temperatures above 27 °C increased the risk of emergency department visits from infectious respiratory diseases (IRD).These results were consistent between men and women in stratified analysis.
Kim et al., 2012	Heat stress (body temperature), systolic blood pressure (SBP), and diastolic blood pressure (DBP)	20 total participants. Men and women, age 63 years and older.	Electronic hygrothermograph used to record indoor temperature and relative humidity at 15-min intervals.	Panel, or repeated measures longitudinal study	Repeated measures ANOVA with random effects to account for clustering of observations within people. Indoor temperature was modeled as a continuous term. Adjusted for sex and age in model for body temperature. Adjusted for sex, age, current smoking, alcohol consumption, and measurement time in model for blood pressure. Stratified estimates by Hypertension status (hypertension versus non-hypertension). Linear regression, Stratified by climate Zone. No adjustment.	Body temperature increased as indoor temperature increased; however, both diastolic and systolic blood pressure decreased as temperature increased. These results remained consistent in individuals living without hypertension but not in individuals living with hypertension.
Li et al., 2018	Thermal discomfort	Total 16,458 participants. Men and women, all ages included.	Dwyer 485 data logger measured indoor temperatures at 15 min intervals.	Panel, or repeated measures longitudinal study	Indoor temperatures were binned into one-degree intervals to estimate change in mean thermal condition vote associated with each one degree Celsius bin of indoor air temperatures.	Thermal discomfort increased as temperature increased across all climate zones.
Lindemann et al., 2017	Physical performance indicated by gait speed, chair rise, balance	81 total participants. Men and women, ages 60 years and older.	Rotronic HL-1D data logger used to measure indoor temperature and humidity at the assessment visit (every four weeks for 30 min). Maximum values used for analysis.	Panel, or repeated measures longitudinal study	Repeated observation, Multilevel linear regression models. No adjustment. Stratified by gait speed.	Gait speed and balance were negatively affected by increases in temperature, but chair rises were not. Habitual gait speed did modify these results.
Loughnan et al., 2015	Thermal comfort	26 total participants in 20 households, Men and women ages 55 years and older.	ibutton temperature loggers used to measure indoor temperature and humidity every 15 min.	Panel, or repeated measures longitudinal study	Linear regression using 2nd order polynomial function. No adjustment, no stratification.	Thermal comfort decreased as indoor temperatures exceeded 26°C.
McCormack et al., 2016	Lung function (Daily symptom scores for breathlessness, rescue inhaler use, Forced Expiratory Volume-1)	69 total participants (former smokers with COPD). Men and women, mean age 69 years (SD = 8).	Maximum daily indoor temperature (°F) measured continuously, methods not reported.	Panel, or repeated measures longitudinal study	GEE models to account for repeated measures. Adjusted for age, sex, education, visit (baseline or 3 or 6 month), and baseline percent predicted lung function (FEV1). Pack-years of smoking were used to account for disease severity for models in which the primary outcome was (FEV1). Also adjusted for PM2.5, NO2, & relative humidity in sensitivity analysis. Explored heterogeneity according to concentrations of air pollutants	Warmer maximum indoor temperatures were associated with worsening of breathlessness, cough, and suptum scale scores, and increases in rescue inhaler use.
O’Lenick et al., 2020	Cause specific mortality related to circulatory diseases, heat related deaths, defined as diagnoses of circulatory, respiratory, renal conditions, and heat stroke/heat exhaustion. Emergency hospital admissions for heat-related conditions, particularly circulatory and respiratory diseases, as well as heat related illnesses.	32,043 total participants. Men and women, all ages	Energy Plus Modeling approaches were used to simulate indoor temperature metrics: Daily maximum or minimum dry-bulb temperature, derived a maximum daily ‘discomfort index’ (DI), defined as the average of maximum dry-bulb and maximum wet-bulb indoor temperatures.	Time stratified case-crossover	Conditional logistic regression. Adjusted for federal holiday, day of the warm season, maximum ambient temperature and maximum ambient dew point temperature (in degrees Celsius, modeled as cubic polynomials). Stratified results by sex, age group, race, census block % Black, % living in poverty, % living alone.	Increases in temperature were associated with mortality and morbidity outcomes, especially for circulatory causes and any heat related deaths. Associations were stronger among adults older than age 65. Associations with emergency hospital admissions were stronger among African Americans.
Quinn and Shamman, 2018	Reports of symptoms of heat illness, temperature and humidity comfort perceptions, sleep quality	40 households (mean 2.3 participants per household). Men and women, ages 2–90 years.	Maxim Integrated DS1923 Hygrochron iButton sensors used to record hourly indoor temperatures	Panel or longitudinal repeat measures	Mixed-effects cumulative or binomial logistic regression models with a random intercept for each household to account for the correlation of effects within households. Adjusted for individual person in household, number of surveys answered, vapor pressure.	Measured indoor temperatures were associated with self-reported sleep problems; Positive but not statistically significant associations between high indoor temperatures and symptoms of heat illness.
Sutton-Klein et al., 2021	Self-rated general health, self-reported “poor health” based on presence of chronic illnesses	74,736 total participants. Men and women ages 16 years and older.	Digital thermometer with a probe used to measure indoor temperatures at the time of survey.	Cross-sectional	Logistic regression models, weighted using survey-provided weights. Adjusted for age & gender in main model, as well as household size, tenure, socioeconomic classification of occupation, income, education, housing variables, SES variables. Stratified by tertiles of outdoor temperature.	Increases in temperature were associated with higher odds of poor self-reported health. These results remained consistent across tertiles of outdoor temperature.
Tartarini et al., 2017	Agitated behavior	325 total participants. Men and women ages > 60 years.	iButton data sensors used to record dry bulb temperature every 15 min.	Panel, longitudinal repeat measures study	Mixed linear regression models. Indoor temperature modeled using a second degree polynomial term. No adjustment, no stratification.	Agitated behavior scores were increased when indoor temperatures increased (and decreased) from 22.6 °C. Cumulative exposure to temperatures >26 °C or < 20 °C. were associated with higher agitated behavior scores.
Teyton et al., 2022	Physical symptoms: cramps, headaches, dry mouth, fatigue, thirst, less frequent urination Mental health symptoms: depression, anxiety	277 total participants. Men and women ages ≥ 60 years.	HOBO sensors recorded dry bulb indoor temperature and humidity every 10 min. Also derived the humidex using the following equation: T + 0.5555(RH × 10.13 × e^^^13.7-(−5,120/273 + T))−10	Panel or longitudinal repeat measures	Generalized estimating equations (GEEs) Poisson distributed models. Modeled temperature as a linear term, and also as natural cubic spline term. Models adjusted for study year, self-reported health status, living alone, sex, age, education, and income. Stratified analyses by preexisting conditions (diabetes, cardiovascular, respiratory, kidney, neurological diseases, cancer)	Increases in temperatures were associated with higher risk of dry mouth, fatigue, thirst, less frequent urination, and trouble sleeping.
Uejio et al., 2016	Distress calls related to respiratory cases and cardiovascular cases	1100 total participants (338 respiratory cases, 291 cardiovascular cases, 471 controls). Men and women. All ages were included, ages ranged from 0 to 98 years old.	HOBO sensors recorded indoor temperature and humidity at a single time point. Indoor heat index was also calculated.	Case-control	Generalized linear (Binomial) models, used to estimate Odds Ratios. Adjusted for time of day and air pollution (ozone and PM_2.5_).	Warmer indoor temperatures associated with higher odds of distress calls for respiratory cases. Odds of cardiovascular cases were similar between cases and controls.
Uejio et al., 2022	Respiratory illness and diabetes	914 total participants: 90 diabetes cases, 126 respiratory cases, 698 controls. Men and women. All ages included, ages ranged from 0 to 10.	HOBO sensors recorded indoor temperature and humidity at a single time point. Indoor heat index was also calculated.	Case-control	Generalized additive models, binomial with a logit link. Temperature modeled using thin plate spline terms. Adjusted for age, sex, comorbidities, outdoor air temperature, wind speed, air pollution ozone and PM2.5.	Odds of diabetes or respiratory distress cases increased non-linearly in association with higher indoor heat index values.
van Loenhout et al., 2016	Annoyance by heat, breathing discomfort, fatigue, annoyance by heat at night and sleep disturbance	113 total participants. Men and women, older population (mean age 73.8 years, SD = 7.5)	iButton Hygrochron used to record indoor temperature and humidity, frequency of recording unspecified.	Cross-sectional	Generalized Estimated Equation (GEE) Poisson log-linear models assuming exchangeable correlation structures, accounting for clustering of observations within individuals.	Increases in temperature were associated with annoyance due to heat and sleep disturbance.
Vellei et al., 2017	Thermal comfort, thermal sensation	55 households. Men and women, all ages. All households had at least one resident who was: over age 65, disabled, had a long term illness.	DS 18B20 sensors recorded indoor air temperatures every 5 min. Indoor temperature measures were used to calculated dT, defined as the difference between room temperature and comfort temperature using the following formula: dT = Troom − Tcomf where Tcomf = 0.33*Trm + 18.8	Cross-sectional	Logistic regression models. No adjustments, no stratification.	Thermal discomfort decreased when indoor temperatures exceeded the comfort temperature. Results suggested that the adaptive model slightly underestimates occupants’ thermal discomfort.
Wei et al., 2022	Thermal comfort	Spring months: 526 participants, Summer months: 609 participants, Men and women. The mean age for the spring subset was 24.2 years, and the mean age of the summer subset was 23.8 years.	Thermal comfort data logger used to measure indoor temperature; Temperature and humidity recorder (TR-72 U), globe temperature recorder (HQZY-1) recorded at a single time point.	Cross-sectional	Linear regressions, no adjustments. Stratified by season (spring and summer).	Increases in temperature were associated with thermal discomfort and thermal acceptance.
Williams et al., 2019	Mean hourly heart rate and mean hourly galvanic skin response	51 total participants. Men and women, older population mean age 65 years.	Undisclosed monitors were used measure indoor dry-bulb temperature	Cross-sectional	Generalized additive mixed models.	Increases in temperatures were associated with higher mean hourly heart rate and mean hourly galvanic skin response.
Yadeta et al., 2022	Thermal comfort	430 total participants. Men and women, age 18 years and older	Indoor air temperature, relative humidity, globe temperature measured using an handheld measurements “AcuRite” digital thermometer recorded 6 times per day.	Cross-sectional	Linear regression, no adjustments and no stratification.	Increases in temperature were associated with greater thermal discomfort.
Yang et al., 2022	Thermal sensation vote	141 total participants. Men and women, all ages.	Indoor temperature, globe temperature measured in °C using using the TH22R-EX data sensor, frequency not specified.	Cross-sectional	Linear regression models, no adjustments. Stratified by city, town, and rural status.	Warmer indoor operative temperature associated with higher thermal sensation vote (i.e., greater discomfort).
Zhang et al., 2018	Thermal sensation vote	24 total participants. Men and women of university student age (mean age 19.7).	Indoor temperature measurement tool was undefined.	Cross-sectional	Linear regression models, no adjustments.	As indoor operative temperature increased, thermal sensation vote while participants were awake and asleep also increased.

1. For detailed information on extracted estimates refer to Supplemental Table 2.

°C = degrees Celsius.

°F = degrees Fahrenheit.

Pm 2.5 = particulate matter 2.5 μg/m^3^.

O3 = ozone g/N m^3^.

NOx = Nitrogen oxide ppm.

**Table 3:** Temperatures cut points used to define heat exposure found in included studies or empirically quantified indoor temperature thresholds.

	Authors, year	Health outcome(s)	Thresholds	How the threshold was determined
*A priori* cut points	Ahrentzen et al., 2016	Sleep quality, emotional health, general health/quality of life, and thermal comfort	How often unit temperatures exceeded ASHRAE standard of 27.2 °C.	ASHRAE-55 in year 2010.
	Beckmann et al., 2021	Subjective heat stress	How often unit temperatures exceeded CIBSE standard of 24 °C.	Chartered Institution of Building Services (CIBSE) threshold were used by the authors which suggests that temperature in bedrooms should not exceed 26 °C for>1 % of the annual occupied hours because sleep quality drops for temperatures above 24 °C.
	Gronlund et al., 2022	Cognitive function and sleepiness	How often unit temperatures exceeded of 22 °C	Based on previous literature on cognitive outcome/sleepiness and indoor temperature.
	Tartarini et al., 2017	Agitated behavior scores	Cumulative percentage of time exposed to temperature < 20 °C or > 26 °C	International Organization for Standardization (IOS) 7730:2005.
Empirically quantified temperature thresholds	Cedeño Laurent et al., 2018	Cognitive function	22 °C	Estimated threshold by cubic spline terms, identification of the minimum temperature after which there was a decrease in cognitive function predicted from fitted environmental exposure models.
	Hansen et al., 2022	Thermal sensation vote	28 °C	Estimated threshold by stepwise linear regression to regress occupants’ thermal sensation votes against personal parameters.
		Perception of health	18–24.3 °C	Estimated threshold by stepwise linear regression to regress occupants’ perception of health being very good against personal parameters.
	Li et al., 2018	Thermal sensation vote	18–26 °C	Estimated thresholds across climate zones, by plotting the mean thermal sensation vote across temperatures and observed that mean thermal sensation vote began to be negatively affected outside the temperature range from 18 °C to 26 °C.
	Lindemann et al., 2017	Physical performance	27.9 °C	Modeled indoor temperature as a categorical variable in 5 bins (<22 °C, 22–23.9 °C, 24–25.9 °C, 26–27.9 °C, >27.9 °C), and found that physical performance was most affected for temperatures >27.9 °C bin.
	Loughnan et al., 2015	Thermal comfort	26.6 °C	The temperature at which the linear regression trend line crossed the y axis and the value for thermal comfort was 0.
	Tartarini et al., 2017	Agitation behavior scores	22.6 °C	Modeled indoor temperature using a second-degree polynomial term and identification of the minimum temperature after which agitation scores increased.
	Teyton et al., 2022	Trouble sleeping	20 °C	Modeled indoor temperature using natural cubic splines, and estimated/plotted the marginal probabilities of observing the health symptom across the temperature distribution. Identified the value after which the probability of symptom reporting increased more rapidly.
		Less urination	22 °C	
		Thirst	18 °C	
		Dry mouth	24 °C	
	Uejio et al., 2016	Cardiovascular distress calls	26 °C	Modeled indoor temperature as a categorical in 3 bins (≥25, 26, and 27 °C) and found that distress calls were most affected in the temperatures >26 °C.
	Uejio et al., 2022	Diabetes related calls Respiratory distress related calls	21.1 °C 24.6 °C	Thin-plate regression spline illustrating the relationship between the log odds of a diabetic case/respiratory distress and indoor heat indices. Also introduced thresholds between the 15th-85th percentile (0.1 °C increments), to identify the value associated with the minimum un-biased risk estimator.
	Wei et al., 2022	Thermal sensation vote:	Thermal sensation vote: Spring (17.9° C) Summer (26.1 °C)	The threshold was determined through regression analysis of the plotted thermal sensation vote to identify the temperature at which the value was equal to or very close to 0.
		Acceptable temperature	The acceptable temperature range: 90 % acceptance is between 19.2 °C and 27.0 °C, and the lower limit for 80 % acceptance is 17.5 °C in spring. The upper limit is 27.7 °C and is 28.9 °C respectively for 90 % and 80 % acceptance in summer.	Seasonal polynomial curves of percentage dissatisfied against indoor operative temperature were extrapolated.
		Thermal preference (percentage dissatisfied)	Spring (23.2 °C) Summer (25.6 °C)	Regression curves of ‘Cooler’ and ‘Warmer’ were obtained, and the threshold temperature was at the intersection of the two curves.
	Yadeta et al., 2022	Thermal sensation vote	20.4 °C	The threshold was determined through regression analysis of the plotted thermal sensation vote to identify the temperature at which the value was equal to or very close to 0.
	Yang et al., 2022	Thermal sensation vote	City (24.8 °C) Town (20.4 °C) Rural (18.2 °C)	The threshold was determined through regression analysis of the plotted thermal sensation vote to identify the temperature at which the value was equal to or very close to 0.
	Williams et al., 2019	Mean hourly heart rate	24 °C	The threshold was determined through visual analysis of the graphed linear regression to identify the temperature at which there was an uptick in adverse outcomes.
		Mean hourly galvanic skin response	24 °C	
	Zhang et al., 2018	Sleep	22.2–28.8 °C	The threshold was determined through regression analysis of the plotted thermal sensation vote to identify the temperature at which the value was equal to or very close to 0.
		Wakefulness	21.1–27.3 °C	

## Data Availability

No data was used for the research described in the article.

## HIGHLIGHTS

- We conducted a scoping review of studies linking warm residential indoor temperatures and health or well-being outcomes.
- Indoor temperature exposure was assessed using sensors, thermometers, data-driven models, and physics-based simulations.
- Safe maximum temperature thresholds ranged from 18 °C to 35 °C, but varied by specific health outcomes.
- Most studies found warmer indoor temperatures were linked to adverse health, well-being, or thermal comfort outcomes.

## Appendix A. Supplementary data

Supplementary data to this article can be found online at https://doi.org/10.1016/j.scitotenv.2025.179377.
