# Supplementary material for: Residential indoor temperatures and health: A scoping review of observational studies
Source: Sci Total Environ. Author manuscript; Available in PMC 2026 Apr 18. (PMC13090820; doi:10.1016/j.scitotenv.2025.179377)
Supplement: Supplemental file [file NIHMS2165438-supplement-Supplemental_file.docx]

| **Supplement Table 1**: Search terms and advanced searchers for each of the four databases and respective registers. | |
| --- | --- |
| Database Name | Search Strategy |
| Web of Science | ((((AB=(indoor temperature)) NOT AB=(indoor air quality )) OR AB=(indoor heat)) OR AB=(residential indoor heat )) OR AB= (indoor heat-health thresholds) and Article (Document Types) AND (((((((((((((((((((((AB=(hospital admission)) OR AB=(hospitalization)) OR AB=(emergency department visit)) OR AB=(respiratory)) OR AB=(cardiovascular)) OR AB=(kidney function)) OR AB=(kidney disease)) OR AB=(renal)) OR AB=(mortality)) OR AB=(cognition)) OR AB=(human comfort)) OR AB=(heat stress)) OR AB=(dehydration)) OR AB=(morbidity)) OR AB=(general health)) OR AB=(life expectancy)) OR AB=(heat exhaustion)) OR AB=(hyperthermia)) OR AB=(patient admission)) OR AB=(heat related illness)) OR AB=(birth outcomes)) OR AB=(maternal health) and Article (Document Types) |
| Global Index Medicus | (ab:(indoor temperature)) OR (ab:( indoor heat-health thresholds)) OR (ab:(indoor heat)) OR (ab:(residential indoor heat)) AND (ab:(hospital admission)) OR (ab:(hospitalization)) OR (ab:(emergency department visit)) OR (ab:(respiratory)) OR (ab:(cardiovascular)) OR (ab:(kidney function)) OR (ab:(kidney disease)) OR (ab:(renal)) OR (ab:(mortality)) OR (ab:(cognition)) OR (ab:(human comfort)) OR (ab:(heat stress)) OR (ab:(dehydration)) OR (ab:(morbidity)) OR (ab:(general health)) OR (ab:(life expectancy)) OR (ab:(heat exhaustion)) OR (ab:(hyperthermia)) OR (ab:(patient admission)) OR (ab:(heat related illness)) OR (ab:(birth outcomes)) OR (ab:(maternal health)) |
| OVID Medline | indoor temperature OR indoor heat-health thresholds OR indoor heat OR residential indoor heat AND hospital admission OR hospitalization OR emergency department visit OR respiratory OR cardiovascular OR kidney function OR kidney disease OR renal OR mortality OR cognition OR human comfort OR heat stress OR dehydration OR morbidity OR general health OR life expectancy OR heat exhaustion OR hyperthermia OR patient admission OR heat related illness OR birth outcome OR maternal health |
| Embase | (((('indoor'/exp OR indoor) AND ('temperature'/exp OR temperature) OR 'indoor'/exp OR indoor) AND ('heat'/exp OR heat) OR 'indoor'/exp OR indoor) AND ('climate'/exp OR climate) OR 'building'/exp OR building) AND ('temperature'/exp OR temperature) OR ‘indoor heat-health thresholds’ COMBINED WITH (((((((((((((hospital AND admission OR hospitalization OR emergency) AND department AND visit OR respiratory OR cardiovascular OR kidney) AND  function OR kidney) AND disease OR renal OR mortality OR cognition OR human) AND comfort OR thermal) AND comfort OR heat) AND stress OR dehydration OR morbidity OR general) AND health OR life) AND expectancy OR heat) AND exhaustion OR hyperthermia OR patient) AND admission OR heat) AND related AND illness OR death OR birth) AND outcomes OR maternal) AND health) |

| **Supplemental Table 2**. Specified health outcomes in each subsection presented in **Figure 3**. | | |
| --- | --- | --- |
| Main Outcome | Collapsed Outcome | Specified Outcome and Respective Citation |
| Physical Outcomes |  |  |
|  | Worsened general health | general health (Ahrenzten et al., 2016; Hansen et al., 2022; Sutton-Klein et al., 2021), physical performance (Lindemann et al., 2017), headaches (Teyton et al., 2022), cramps (Teyton et al., 2022) |
|  | Cardiovascular distress | pulse rate (Goldberg et al., 2015), cardiovascular disease- related emergency department visits (Jung 2020), distress medical calls related to cardiovascular cases (Uejio et al., 2016; Uejio et al., 2022), circulatory mortality (O’Lenick et al., 2020), circulatory hospitalizations (O’Lenick et al., 2020), higher blood pressure (Barnett et al., 2007; Goldberg et al., 2015; Kim et al., 2012) and mean hourly heart rate ( Williams et al., 2019), distress calls related to diabetes (Uejio et al., 2022) |
|  | Respiratory Distress | non-infectious respiratory diseases (Jung et al., 2021), lung function (McCormack et al., 2016), distress medical calls for respiratory cases (Uejio et al., 2016, Uejio et al., 2022), breathing discomfort (vanLoenout et al., 2016), shortness of breath (Goldberg et al., 2015), oxygen saturation (Goldberg et al., 2015), acute respiratory illness (Han et al., 2020) |
| Intermediate Outcomes |  |  |
|  | Poor Sleep | interrupted or impaired sleep (Ahrenzten et al., 2016, Gronlund et al., 2022; Quinn et al., 2017; van Loenhout et al., 2016; Zhang et al., 2018) |
|  | Lower Cognitive Function | impaired cognitive function (Cedeño Laurent et al., 2018; Gronlund et al., 2022), agitation related dementia (Tartarini et al., 2017), fatigue (van Loenhout et al., 2016) |
|  | Heat Stress | subjective heat stress (Beckman et al., 2021), objective heat stress (Kim et al., 2012), annoyance by heat (van Loenhout et al., 2016) annoyance by heat at night (van Loenhout et al., 2016), mean hourly galvanic skin response (Williams et al., 2019), and dry mouth (Teyton et al., 2022) |
|  | Thermal Discomfort | thermal discomfort (Hansen et al., 2022; Loughnan et al., 2015; Vellei et al., 2017), poor thermal sensation (Li et al., 2018; Wei et al., 2022; Yadeta et al., 2022; Yang et al., 2022), poor thermal preference ( Wei et al., 2022), poor thermal acceptability (Wei et al., 2022) |
| Mental Health Outcomes |  |  |
|  | Worsened Emotional Health | increased emotional distress (Ahrentzen et al., 2016) |
|  | Depression/ Anxiety | anxiety and depressive symptoms (Teyton et al., 2022). |

| **Supplemental Table 3.** Detailed extraction of estimates from results included in this systematic review | | | | | | |
| --- | --- | --- | --- | --- | --- | --- |
| **Author, Year** | **Objective** | **Outcome** | **Study Population** | **Temperature** | **Study Design and Analysis** | **Results** |
| Ahrentzen, 2016 | To ascertain if energy retrofits in affordable housing for older adults correspond with improved health and comfort for its residents. | Health conditions of resident, including sleep, emotional health, general health/quality of life , and thermal comfort | 57 total participants. Men and women, ages ranged from 62-92 years old. | Indoor temperature was measured in absolute air temperature (Degrees Celsius) using HOBO U-10-001 data sensors. | Panel Study, Fixed effects models with ordinal regression models were used to examine whether the changes of temperature that exceeded 27 ^o^C between P1 and P3 in a resident’s apartment contributed to changes in that resident’s reported health over the same time period. Models were adjusted for floor level of participant’s unit. | Beta estimates and p-value for temperatures that exceed 27 °C , comparing P1 and P3 quality of life: 3.179 (<0.01), emotional distress: -2.085 (<0.05), hours of sleep 2.150 (<0.05) |
| Barnett, 2007 | To estimate the association between systolic blood pressure and season, indoor and outdoor temperature, and short-term trends in outdoor temperature. | Systolic blood pressure | 115,434 total participants. Men and women, ages ranged from 35–64 years old. | Indoor temperature was obtained via undefined methods | Cross-sectional study, Bayesian hierarchical model including, age, sex, and type of sphygmomanometer. In addition, trends in temperature over time were estimated in each population using a smooth nonlinear model of outdoor temperature, while also accounting for seasonal change. | Beta estimate for entire population associated with an increase in mean systolic blood pressure for every 1 °C increase in indoor temperature -0.31mmHg ( -0.44, -0.19). |
| Beckmann, 2021 | To better understand overheating thresholds and to compare them with thresholds for subjective heat stress during heatwaves. | Subjective heat stress | 427 households, Men and Women, all ages, including those under 18 years of age | Indoor temperature (type undefined) was measured in degrees Celsius using loggers (Elitech RC-5). | Paired t-test; Mann-Whitney-U test, and Regression analysis, Mean heat stress was stratified by nonvulnerable groups, people who identified as living alone, people with chronic diseases, and people with increased exposure to heat (living on a high floor or living in a hot local climate zone) | The following means and standard deviations are for subjective heat stress.  Indoor temperature < or = 24.9 °C for full population: 2.75 (1.33)  Indoor temperature > 24.9 °C for full population: 3.23 (1.14)  Indoor temperature < or = 25.7 °C for people younger than 65 without chronic condition: 2.94 (1.06)  Indoor temperature > 25.7 °C for people younger than 65 without chronic condition: 3.35 (1.02)  Indoor temperature < or = 24.2 °C for people living alone: 1.75 (0.96)  Indoor temperature > 24.2 °C for people living alone : 3.17(1.24)  Indoor temperature < or = 26.7 °C for people living with chronic condition: 3.00(1.41)  Indoor temperature > 26.7 °C for people living with chronic condition: 3.56(1.13)  Indoor temperature < or = 26.3 °C for people living on high floor (above 4th) or living in most affected local climate zone : 2.74(1.37)  Indoor temperature > 26.3 °C for people living on high floor (above 4th) or living in most affected local climate zone : 3.38(1.14)  Indoor temperature < or = 26.2 °C for group with the highest slope of temperature regression: 2.57(1.4)  Indoor temperature >26.2 °C for group with the highest slope of temperature regression: 3.49(1.14)  Linear regression results  Unstandardized Beta coefficient 0.271 (SE= 0.1)  Standardized Beta coefficient 0.195 (t=2.705) |
| Cedeño Laurent, 2018 | To examine relationships between indoor environment-al conditions, heat exposures, sleep, and cognitive function between young adults living in central AC and non-AC residence halls on a university campus before, during, and after a heat wave during the summer of 2016. | Cognitive function performance tests via the Stroop color-word test (STROOP) to assess selective attention/processing speed and a visual addition/subtraction test (ADD) to evaluate cognitive speed and working memory. | 44 total participants. Men and women, ages ranged from 18 to 29 years old | Indoor temperature was measured by dry bulb temperature (degrees Celsius) using an undefined indoor quality monitor. | Cohort; Generalized additive mixed models were used to estimate the individual effects of indoor  environmental parameters on cognitive function. Student ID, nested within building type, was treated as a random effect to account for differences between individuals. Environmental exposures to maximum indoor temperature, mean noise, mean absolute humidity, and mean CO_2_ concentrations were computed for the overnight period prior to each cognitive test and included into a single model for each cognitive function outcome of interest.  Nonlinear effects of continuous variables were evaluated using penalized splines; only variables that exhibited a significant nonlinear effect were kept in the model as spline terms. Covariates included hydration (glasses per day less than the median=1; otherwise =0), caffeine intake, time from waking up to taking the test (time in hours). | Fixed effect estimates from indoor temperature (Maximum indoor temperature during the sleep period every 1 °C)  Reaction time, assessed using ADD: 0.01 (0.003, 0.02)  Throughput, assessed using ADD: -0.04 (-0.08, -0.002)  Inhibitory control time, assessed with STROOP: 0.03 (0.005, 0.05)  Fixed effect estimates from indoor temperature (Maximum indoor temperature during the sleep period (degrees Celsius) by quartiles.  Reaction time, assessed with STROOP:  Q2: -0.09 (-0.19, 0.01)  Q3: 0.02 (-0.15, 0.10)  Q4: 0.23 (0.06, 0.38)  Throughput, assessed with STROOP:  Q2: 0.17 (-0.05,0.39)  Q3: -0.02 (-0.28, 0.24)  Q4: -0.32 (-0.64, 0.01) |
| Goldberg, 2015 | To estimate among persons with heart failure the extent to which daily exacerbations in essential signs, symptoms, and physiologic indicators were associated with daily variations in health-related factors, and concentrations of air pollution and meteorological indicators measured at participants homes. | Oxygen saturation (%), pulse rate, systolic blood pressure (mm Hg), Diastolic blood pressure (mmHg), Perception of health compared with other people same age (visual analogue scale), and shortness of breath at night (visual analogue scale) | 55 total participants. Men and Women Ages over 35 years old | Indoor temperature (type not indicated) was measured in (degrees Celsius) using HOBO data sensors | Panel study; Mixed random effects model using restricted maximum likelihood estimation to account for the lack of independence by making each subject equivalent to a cluster (random effects on each subject), thus allowing for estimation of within-subject and between-subject variances. Used an autoregressive model of order one to account for correlation between days.  Day in study (1 to 56 maximum days), month on study; possible infections the day before (binary variable); consumption of salt the day before; number of cups of liquid consumed the day before. Adjusted for air pollutants (CO, fine particles - both indoor and outdoor). | Estimates for mean change for interquartile range increase (2.86) for lag day 0  Oxygen saturation: -0.140 (-0.031, 0.047)  Oxygen saturation: -0.142 (-0.254, -0.030)  Oxygen saturation: -0.176 (-0.300, -0.052)  Mean change in pulse rate : 0.005(-0.636, 0.647)  Mean change in pulse rate : 0.044(-0.596, 0.684)  Mean change in pulse rate : 0.158 (-0.609, 0.926)  Mean change in diastolic blood pressure (mmHg) :-0.516 (-1.214, 0.183)  Mean change in systolic blood pressure (mmHg): -1.462 (-2.411, -0.512)  Mean change in self-rated health: -0.536 (-1.135, 0.064)  Mean change in shortness of breath at night: -1.204b(-2.075, -0.333) |
| Gronlund, 2022 | To study short-term associations between indoor temperatures and cognitive function and daytime sleepiness in low-income residents of Detroit, MI | Cognitive function [(WLL: word list listening, WLD: word list delayed)] and sleepiness | 18 total participants. Men and Women ages ranged from 28-77 years old with a median age of 58 years | Indoor temperature was measured in absolute temperature (degrees Celsius) using HOBO data sensors. When temperature measurements were missing, researchers simulated temperature by mathematical equation: apparent temperature (AT) = - 2.653 + (0.994 x temperature) + 0.0153 x (dew point)^2^ | Cohort, repeated measures fixed effects analyses, which included a dummy variable for each participant in a linear regression of temperature on cognitive outcome/sleepiness., Controlled for individual participant within repeated-measures analysis. | Beta coefficients and 95% CI of point change for full population:  2 °C decrease in AT 22 °C for sleepiness score (before the survey): 0.5 (-0.0, 1.1)  2 °C decrease in AT 22 °C for sleepiness score at lag hour 10-20: -0.3 (-0.5, -0.0)  2 °C increase in AT >22 °C at lag hour 12 (before the survey) WLL score: 0.1 (0.0, 0.3)  2 °C increase in AT >22 °C at lag hour 12 (before the survey) WLD score: 0.1 (0.0, 0.3)  2 °C increase in average nighttime AT >22 °C (night before the survey)  WLD score: 0.7 (0.1, 1.4)  2 °C increase in average nighttime AT (night before the survey) WLL score: 0.4 (0.0, 0.7) |
| Han, 2020 | To investigate the impact of household environmental factors, including temperature, relative humidity, and absolute humidity on acute respiratory illness. | Acute respiratory illness (ARI) | 285 total participants. Men and women, 65 and older; Median age at recruitment: 77 years | Indoor temperature (degree Celsius) (type undefined) was measured using HOBO sensors. | Time stratified case-crossover design; conditional logistic regression models.  Calculated excess risks (ERs) of ARI associated with per-unit increase indoor temperature.  Adjusted for precipitation, PM_2.5_, and O_3_ may modify the effect estimates of environmental factors for Cumulative effects up to 6 lag days (lag0–lag06) | Exact estimates unable to be extracted from figures presented in article.  Overall, the researchers found a weak association between indoor temperature and acute respiratory illness. |
| Hansen, 2022 | To determine links between the indoor thermal environment of housing and self-reported health and wellbeing in older people in metropolitan and regional areas of South Australia. | Self-reported general health and quality of life (questions adopted from the EQ-5D-5L questionnaire) | 303 total participants. Men and Women, ages ranged from 61-98 years old | Indoor temperature (type undefined) was measured using data sensors (CCS811 sensor). | Cross-sectional, linear regression adjusted for age and sex | Exact estimates unable to be extracted from figures presented in article.  Overall, the researchers found the health of older occupants worsened when indoor temperatures were above 28 °C. |
| Jung, 2020 | To investigate the cumulative effects of high indoor temperature exposure on the risk of cardiovascular  diseases. | Cardiovascular disease-related emergency department visits | 30 total households Men and Women aged older than or equal to 65 years of age | Indoor temperature was measured in hourly temperature (degrees Celsius) based on estimation using a prediction model which created cumulative degree hours. The prediction model included hourly levels of indoor and outdoor temperature and relative humidity, land surface  temperature, normalized difference vegetation index (NDVI), building characteristics, occupants' behavior, and electricity consumption data compiled from  30 households. | Case-crossover; Conditional logistic regression, Adjustment for air pollution (PM _2.5_, NO_x_, O_3_), time of emergency room visit, day of week, and holiday. Estimates were stratified by age and sex | Estimates for cumulative degree hours for cardiovascular disease emergency room department visits for older adults for full population and stratified by sex and age  Full population  cardiovascular disease–related ED visits at 27 °C RR: 1.145 (1.003, 1.307)  cardiovascular disease–related ED visits at 28 °C RR: 1.078 (1.001, 1.161)  cardiovascular disease–related ED visits at 29 °C RR: 1.035 (1.001, 1.071)  cardiovascular disease–related ED visits at 30 °C RR: 1.013 (1.007, 1.020)  cardiovascular disease–related ED visits 31 °C RR: 1.082 (1.056, 1.109)  Stratified by Men  cardiovascular disease–related ED visits at 27 °C RR: 1.183 (1.006, 1.392)  cardiovascular disease–related ED visits at 28 °C RR: 1.133 (1.004, 1.279)  cardiovascular disease–related ED visits at 29 °C RR: 1.082 (1.004, 1.166)  cardiovascular disease–related ED visits at 30 °C RR: 1.032 (1.018, 1.047)  cardiovascular disease–related ED visits at 31 °C RR: 1.197 (1.132, 1.266)  Stratified by Women  cardiovascular disease–related ED visits at 27 °C RR: 1.159 (1.004, 1.337) cardiovascular disease–related ED visits at 28 °C RR: 1.130 (1.007, 1.269)  cardiovascular disease–related ED visits at 29 °C RR: 1.081 (1.003, 1.165)  cardiovascular disease–related ED visits at 30 °C RR: 1.035 (1.021, 1.050)  cardiovascular disease–related ED visits at 31 °C RR: 1.197 (1.137, 1.260)  Stratified by age 65-74 cardiovascular disease–related ED visits at 27 °C RR: 1.148 (1.00004, 1.318)  cardiovascular disease–related ED visits at 28 °C RR: 1.144 (1.006, 1.300)  cardiovascular disease–related ED visits at 29 °C RR: 1.098 (1.002, 1.203)  cardiovascular disease–related ED visits at 30 °C RR: 1.027 (1.015, 1.039)  cardiovascular disease–related ED visits at 31 °C RR: 1.141 (1.080, 1.205)  Stratified by age 75-84 cardiovascular disease–related ED visits at 27 °C RR: 1.178 (1.00004, 1.387)  cardiovascular disease–related ED visits at 28 °C RR: 1.135 (1.005, 1.282)  cardiovascular disease–related ED visits at 29 °C RR: 1.063 (1.001, 1.129)  cardiovascular disease–related ED visits at 30 °C RR: 1.035 (1.019, 1.051)  cardiovascular disease–related ED visits at 31 °C RR: 1.221 (1.157, 1.289)  Stratified by age >85  cardiovascular disease–related ED visits at 27 °C RR: 1.222 (1.011, 1.477)  cardiovascular disease–related ED visits at 28 °C RR: 1.193 (1.002, 1.421)  cardiovascular disease–related ED visits at 29 °C RR: 1.018 (1.004, 1.033)  cardiovascular disease–related ED visits at 30 °C RR: 1.042 (1.019, 1.065)  cardiovascular disease–related ED visits at 31 °C RR: 1.264 (1.172, 1.363) |
| Jung, 2021 | To estimate the cumulative effects of indoor temperature exposure by using cumulative degree hours on emergency department visits for infectious and non-infectious respiratory diseases among older adults, and to estimate how these associations are affected by gender. | Infectious and non-infectious respiratory diseases | 231,282 total participants gathered. Men and Women; older than 65 years old | Indoor temperature was measured using prediction models based on indoor/outdoor temperature (degrees Celsius) indoor/outdoor relative humidity (%), atmospheric pressure (mmHg), global solar radiation (Million-Joule/m^2^), wind speed (m/s), and wind direction (o), land surface temperature (^o^C), NDVI, electricity consumption (kilowatt-hour/day), and building characteristics (building structure type: stone or reinforced concrete, building age (year), building level (floor)) between years 2006 and 2014. | Case-crossover; A distributed lag nonlinear model with a quasi-Poisson function was used. Daily concentrations of air pollutant concentrations (NO_x_, PM_2.5_, and O_3_), day of the week, and holiday days were adjusted in the models. Estimates were stratified by gender (male versus female) | Estimates for cumulative degree hours for non-infectious emergency room department visits for older adults for full population and stratified by gender  Full population  non-infectious respiratory disease ER visits at 31 °C RR: 1.124 (1.028, 1.229)  Stratified by Men  non-infectious respiratory disease ER visits at 27 °C RR: 1.276 (1.005, 1.620)  non-infectious respiratory disease ER visits at 28 °C RR: 1.199 (1.000, 1.437)  non-infectious respiratory disease ER visits at 29 °C RR: 1.033 (1.015, 1.052)  non-infectious respiratory disease ER visits at 30 °C RR: 1.198 (1.005, 1.428)  non-infectious respiratory disease ER visits at 31 °C RR: 1.174 (1.046, 1.317)  Stratified by Women  non-infectious respiratory disease ER visits at 27 °C RR: 1.586 (1.005, 2.504)  non-infectious respiratory disease ER visits at 28 °C RR: 1.459 (1.001, 2.126)  non-infectious respiratory disease ER visits at 29 °C RR: 1.366 (1.010, 1.848) |
| Kim, 2012 | To estimate the indoor temperature of low economic housing (dosshouses) and to assess the acute effects of heat stress on body temperature and blood pressure in elderly individuals living in poor housing conditions. | Heat stress (body temperature), systolic blood pressure (SBP), and diastolic blood pressure (DBP). | 20 total participants. Men and women over the age of 63. | Indoor temperature was measured (type undefined) in degrees Celsius using  an electronic hygrothermograph (AE-817CE, B&J, China). | Cross-sectional; Multivariate models, adjusted for age, sex, alcohol status, smoking status, time of measurement. Stratified estimates by hypertension status (hypertension versus non-hypertension) | Beta coefficients and associated standard errors for every 1 °C degree increase in indoor temperature with studied outcomes, stratified by individuals with hypertension versus not.    Full population  Body temperature Beta: 0.205 (SE: 0.025)  Systolic Blood pressure (mmHg) Beta: -1.750 (SE: 1.451)  Diastolic blood pressure (mmHg) Beta: -2.049 (SE: 1.014)  Individuals with hypertension  Body temperature Beta: 0.258 (SE: 0.044)  Systolic Blood pressure (mmHg) Beta: 3.175 (SE: 2.717)  Diastolic blood pressure (mmHg) Beta: - 0.690 (SE: 2.024)  Individuals without hypertension  Body temperature Beta: 0.188 (SE: 0.032)  Systolic Blood pressure (mmHg) Beta: - 3.170 (SE: 1.718)  Diastolic blood pressure (mmHg) Beta: - 1.429 (SE: 1.131) |
| Li, 2018 | To investigate the associations between indoor thermal environment and thermal comfort in residential buildings, and how these associations differed by five different climate  zones. | Thermal comfort | total 16,458 participants. Men and Women, all ages included | Indoor temperature (type undefined) was measured in degrees Celsius using data sensors (Dwyer 485) | Cross-sectional. Linear regressions, Stratified by zone | Beta coefficients for every 1 °C degree increase in indoor temperature with thermal sensation (thermal sensation vote), stratified by city.  Shenyang and Harbin city in SC zone: 0.0976  Xi'an in C zone: 0.094  Chongqing, Wuhan, and Chengdu in HSCW zone: 0.0942  Fuzhou and Guangzhou in HSWW zone: 0.1134  Kumming in M zone:0.0744 |
| Lindemann, 2017 | To estimate the association between indoor temperature and physical performance in the indoor home environment of older adults during the summer of 2015 within and outside of heatwaves. | Physical performance indicated by gait speed, chair rise, balance | 81 total participants. Men and Women, aged 60 years and older | Indoor temperature (type undefined) was measured in degrees Celsius using data logger (HL-1D, ROTRONIC Messgeräte GmbH, Ettlingen, Germany) | Panel study, repeated observation, Multilevel linear regression models. Stratified by gait speed | Mean change and associated with 95% CI every 10 °C degree increase in indoor temperature for studied physical health outcomes  Gait speed Full population : -0.074 (-0.113, -0.034)  adults with initially with higher gait speed: -0.087 (-0.136, -0.038)  adults with initially with lower gait speed: -0.044 (-0.109, 0.021)  Chair rise  Full population: 1.15 (0.58, 1.73) adults with initially with higher gait speed: 0.67 (0.12, 1.23) adults with initially with lower gait speed: 2.03 (0.79, 3.28)  Balance Full population: -1.69 (-3.56, 0.182) adults with initially with higher gait speed: -0.18 (-2.33, 1.98)  adults with initially with lower gait speed: -3.92 (-7.31, -0.52)  Mean change and associated with 95% CI between heatwaves and normal days (difference between heatwaves defined as 3+ days of temperatures higher than 30 °C and other days) and studied physical health outcomes  Gait speed Full population: -0.041 (-0.065, -0.018)  adults with initially with higher gait speed: -0.062 (-0.092, -0.032)  adults with initially with lower gait speed: -0.010 (-0.048, 0.027)  Chair rise  Full population: 0.90 (0.55, 1.24) adults with initially with higher gait speed: 0.57 (0.23, 0.90)  adults with initially with lower gait speed:1.44 (0.72, 2.16)  Balance  Full population: -1.11 (-2.24, 0.01)  adults with initially with higher gait speed: -0.93 (-2.27, 0.41)  adults with initially with lower gait speed: -1.36 (-3.33, 0.61) |
| Loughnan, 2015 | To understand how structural and technological adaptations influenced indoor household temperatures within a cohort of older people living in an Australian town that routinely experiences hot summer weather. | Thermal comfort | 26 total participants in 20 households, Men and Women aged 55 years and older | Indoor temperature measured in degrees Celsius (type undefined) was measured using data sensors (buttons) | Cross sectional, Regressions using 2^nd^ order polynomial function | Exact estimates unable to be extracted from figures presented in article.  Overall, the researchers found a linear relationship as when temperature increased, thermal comfort scores increased indicating worse comfort. |
| McCormack, 2016 | To determine how increases in indoor and outdoor temperature during the warmer months would be associated with increases in daily respiratory symptoms and rescue medication use and decreases in lung function among participants with COPD. To determine how increases in air pollution exposure would modify the effects of temperature, enhancing the detrimental effects of increases in temperature on these daily indicators of COPD morbidity. | Lung function of former smokers with COPD | 69 total participants. Men and Women that were older in age, population with mean 69 years old (SD=8) | Indoor temperature was measured in maximum daily indoor temperature (degrees ^o^F) using undefined methods | Cohort, GEE models to account for repeated measures, age, sex, education, visit (baseline or 3 or 6 month), and baseline percent predicted lung function (FEV1). Pack-years of smoking were used to account for disease severity for models in which the primary outcome was (FEV1). Also adjustment for PM_2.5_, NO_2_, & relative humidity in sensitivity analysis. | Beta coefficients, with 95% CI, for every 10 °F degree increase in indoor temperature with various lung function parameters by lag day  daily maximum temperature (°F ), lag 0, Breathlessness, Cough, and Sputum Scale (BCSS): 0.30 (0.00, 0.59)  daily maximum temperature (°F ), lag 1, Breathlessness, Cough, and Sputum Scale (BCSS): 0.36 (0.01, 0.70)  daily maximum temperature (°F), lag 2, Breathlessness, Cough, and Sputum Scale (BCSS): 0.48 (0.12, 0.85)  daily maximum temperature (°F), lag 3, Breathlessness, Cough, and Sputum Scale (BCSS): 0.10 (-0.27, 0.47)  daily maximum temperature (°F), lag 0, Rescue inhaler use: 0.26 (0.09, 0.42)  daily maximum temperature (°F), lag 1, Rescue inhaler use: 0.17 (-0.02, 0.36)  daily maximum temperature (°F), lag 2, Rescue inhaler use: 0.21 (-0.01, 0.42)  daily maximum temperature (°F), lag 3, Rescue inhaler use: -0.02 (-0.24, 0.20)  daily maximum temperature (°F), lag 0, Lung function (evening FEV1): -0.02 (-0.05, 0.02)  daily maximum  temperature (°F), lag 1, Lung function (evening FEV1): -0.01 (-0.04, 0.03)  daily maximum temperature (°F), lag 2, Lung function (evening FEV1): -0.01 (-0.05, 0.02)  daily maximum temperature (°F), lag 3, Lung function (evening FEV1): -0.02 (0.06, 0.02) |
| O'Lenick, 2020 | To estimate the health impacts of indoor heat and evaluate vulnerability factors | Mortality outcomes: circulatory, & all HEAT-related (basically all a priori associated with heat including circulatory)  morbidity outcomes: emergency hospital admissions for circulatory & all HEAT-related | 32,043 total participants. Men and Women, all ages | Indoor temperature was measured in maximum daily dry-bulb temperature,  Minimum (degrees Celsius) daily dry-bulb temperature (degrees Celsius), and  maximum daily 'discomfort index' (DI) (degrees Celsius): average of max dry-bulb and max wet-bulb temperatures indoors using the following equation DI= 0.5 (dry bulb temperature* wet bulb temperature). All temperature indicators were measured using Energy Plus Modeling approaches | Case-crossover, Conditional logistic regression (matched by strata of census block group, year, month, and weekday), Adjusted for federal holidays, day of the warm season, maximum ambient temperature and maximum ambient dew point temperature (in degrees Celsius, modeled as cubic polynomials), stratified results by sex, age group, race, Black race census block %, Below poverty census block %, Living alone census block % | Odds ratio, with 95% CI for every 5 °C degree increase in maximum daily discomfort index, lag 0-2 (unless otherwise noted) with mortality and hospitalizations stratified by demographic factors  Full population   Max DI, lag 0-2 Heat-related mortality: 1.20 (1.02, 1.40) Circulatory mortality: 1.31 (1.10, 1.57) Heat-related emergency hospital admissions: 1.12 (0.99, 1.26) Circulatory emergency hospital admissions: 1.12 (0.96, 1.30)  MAX dry-bulb temp, lag 0-2 Heat-related mortality: 1.10 (0.99, 1.22) Circulatory mortality: 1.16 (1.03, 1.30) Heat-related emergency hospital admissions: 1.08 (0.99, 1.17) Circulatory emergency hospital admissions: 1.09 (0.99, 1.21)  MIN dry-bulb temp, lag 0-2 Heat-related mortality: 1.22 (0.95, 1.57) Circulatory mortality: 1.37 (1.03, 1.82) Heat-related emergency hospital admissions: 1.09 (0.90, 1.31) Circulatory emergency hospital admissions: 1.02 (0.81, 1.29)  Odds ratios (95% confidence intervals) between 3-d moving averages of maximum DI and health outcomes stratified by individual factors.  Women  Circulatory mortality: 1.16 (0.92, 1.47) Heat-related mortality: 1.17 (0.95, 1.44) Circulatory emergency hospital admissions: 1.08 (0.89, 1.31) Heat-related emergency hospital admissions: 1.14 (0.97, 1.33)  Men  Circulatory mortality: 1.52 (1.17,1.99) Heat-related mortality: 1.23 (0.98, 1.55) Circulatory emergency hospital admissions: 1.17 (0.92, 1.50) Heat-related emergency hospital admissions: 1.09 (0.90, 1.32)  Ages 65-74 Circulatory mortality: 1.55 (1.11, 2.17) Heat-related mortality: 1.41 (1.05, 1.90) Circulatory emergency hospital admissions: 1.14 (0.90, 1.44) Heat-related emergency hospital admissions: 1.11 (0.92, 1.34)  Ages >74 Circulatory mortality: 1.25 (1.01, 1.53) Heat-related mortality: 1.14 (0.95, 1.36) Circulatory emergency hospital admissions: 1.11 (0.91,1.36) Heat-related emergency hospital admissions: 1.13 (0.96,1.32)  White  Circulatory mortality: 1.39 (1.07, 1.81) Heat-related mortality: 1.19 (0.94, 1.49) Circulatory emergency hospital admissions: 0.89 (0.65, 1.20) Heat-related emergency hospital admissions: 0.90 (0.71, 1.15)  African American  Circulatory mortality: 1.41 (1.07, 1.86) Heat-related mortality: 1.36 (1.06, 1.73) Circulatory emergency hospital admissions: 1.34 (1.05, 1.71) Heat-related emergency hospital admissions: 1.24 (1.02, 1.51) |
| Quinn and Shamman, 2018 | To study indoor measured climate & perceived climate in relation to health symptoms in New York City residential environments | Self-reported sleep quality & and reports of symptoms experienced in the household | 40 households (with a mean 2.3 participants per household). Men and Woman, ages ranged from 2-90 with a median age of 28.5 | Indoor temperature (type undefined) was measured ( degrees Celsius) using Maxim Integrated DS1923 Hygrochron iButton sensors.  Perceived temperature  Temperature and humidity perceptions were assessed on a 5-point scale from “very cold/very dry” through “neutral” to “very hot/very humid.” | Cross-sectional study, mixed-effects cumulative or binomial logistic regression models with a random intercept for each household to account for the correlation of effects within households, adjusted for individual person in household, number of surveys answered | Beta coefficients [odds ratios] for sleep rates “worse than usual" vs “better than usual"  for indoor temperature from mixed-effects logistic regression models  summer indoor temp on prior day, 1 °C increase:0.83 [2.28]  perceived summer indoor temp on prior day, 1-unit increase on 5-pt scale: 1.25 [3.47]  Coefficients [Odds Ratios] of heat illness (combining serious illness & warning signs; only cases occurring at home) reports predicted by measured and perceived conditions, derived from mixed-effects logistic models, adjusted for number of household members.  measured summer indoor temp today: 0.17 [1.19]  measured summer indoor temp last 3 weeks: 0.24 [1.27]  perceived summer indoor temp today, 1-unit increase on 5-pt scale: 0.88 [2.41]  perceived summer indoor temp comfort, today, 1-unit increase on 5-pt scale: 2.01 [7.46]  perceived summer indoor temp LAST 3 weeks, 1-unit increase on 5-pt scale: 1.26 [3.53]  perceived summer indoor temp comfort, LAST 3 weeks, 1-unit increase on 5-pt scale: 1.64 [5.16]  Coefficients and incidence rate ratios [IRR] from Poisson models of heat illness (combining serious illness & warning signs; only cases occurring at home) in the household predicted by measured mean indoor temperature over the summer season; and by average perceptions over the season.  measured summer indoor temperature (mean over season): -0.34 [0.71]  perceived summer indoor temperature (mean over season), 1-unit increase on 5-pt scale: 1.21 [3.35]  perceived summer indoor temperature comfort (mean over season): 2.07 [7.92] |
| Sutton-Klein, 2021 | To study associations between indoor temperature and self-rated health. | Self-rated health | 74,736 total participants. Men and Women aged 16 years and older | Indoor temperature (type undefined) was measured in degrees Celsius using  using a digital thermometer with a probe | Cross-sectional, Logistic regression, weighted using survey-provided weights, Adjusted for age & gender in main model, then other covariates in subsequent models: household size, tenure, SES of occupation, income, education, 'housing variables', 'SES variables', and stratified by outdoor temperature | Fully adjusted odds ratios and 95% CI for self-rated poor health and indoor temperature (1 °C increase) for full population and stratified by outdoor temperature  Full population  1.02 (1.01, 1.03)  lowest tertial of outdoor temp (<7 °C)  1.04 (1.02, 1.05)  middle tertial of outdoor temp (7-12.8 °C)  1.02 (1.00- 1.04)  highest tertial of outdoor temp (>12.9 °C)  1.01 (0.99- 1.02) |
| Tartarini, 2017 | To gather quantitative evidence regarding the possible correlation between indoor air temperature and agitation of people with dementia. | Agitative behavior due to cognitive dementia measured by the Cohen-Mansfield Agitation Inventory (CMAI) | 325 total participants. Men and women ages > 60 years. | Indoor temperature (as dry bulb temperature) was measured and logged using 24 iButton data sensors. | Cohort study, Fixed regression analyses: The first model examined the correlation between average indoor air temperature and CMAI Total Frequency scores. The second model examined the specific impact that cumulative exposure to temperatures outside the comfort temperature range had on agitated behaviors. | Agitated behaviors was significantly correlated with the amount of hours that residents were exposed to temperatures higher than 26 ^o^C and lower than 20 ^o^C  %time outside comfort range  Beta estimate: 0.0000646, standard error: 0.0000182. |
| Teyton, 2022 | To assess the relationship between indoor heat and various physical and mental health symptoms | Physical symptoms: cramps, headaches, dry mouth  Mental health symptoms: depression, anxiety | 277 total participants. Men and women ages >=60 years. | Indoor temperature was measured by dry-bulb temperature ( degrees Celsius ) using HOBO sensors | Cohort Study, generalized estimating equations (GEEs) for the longitudinal data across the three exposure periods (T1, T2, T3) with exchangeable correlation matrix, using Poisson regression models to estimate RRs & CIs. Models adjusted for study year (2017; 2018), self-reported health status (reported existing health issues; did not report any existing health issues), living alone (dichotomous based on continuous number of individuals living in household), sex (female; male), age (continuous), education (primary school; high school; college/university), and income (insufficient financial resources; sufficient financial resources. Results stratified by pre-existing health conditions | Risk ratio and 95 CI for indoor temperature (°C) and self-rated health items stratified by outdoor temperature.   Fatigue T2 (28-30  ^o^C outdoor) vs. T1 (18-22 ^o^C outdoor): 1.78 (1.36, 2.34) Fatigue T3 (30-33 ^o^C outdoor) vs. T1 (18-22 ^o^C outdoor) : 2.29 (1.76, 2.97) Headache T2 (28-30 ^o^C outdoor) vs. T1 (18-22 ^o^C outdoor): 1.12 (0.77, 1.64) Headache T3 (30-33 ^o^C outdoor) vs. T1 (18-22 ^o^C outdoor): 1.25 (0.84, 1.84) Cramps T2 (28-30 ^o^C outdoor) vs. T1 (18-22 ^o^C outdoor): 1.29 (1.00, 1.67) Cramps T3 (30-33 ^o^C outdoor) vs. T1 (18-22 ^o^C outdoor): 1.37 (1.06, 1.77) Dry mouth T2 (30-33 ^o^C outdoor) vs. T1 (18-22 ^o^C outdoor): 2.00 (1.40, 2.86) Dry mouth T3 (30-33 ^o^C outdoor) vs. T1 (18-22 ^o^C outdoor): 2.48 (1.75, 3.50) Trouble sleeping T2 (30-33 ^o^C outdoor) vs. T1 (18-22 ^o^C outdoor): 1.44 (1.01, 2.05) Trouble sleeping T3 (30-33 ^o^C outdoor) vs. T1 (18-22 ^o^C outdoor): 2.24 (1.59, 3.15) Lightheadedness T2 (30-33 ^o^C outdoor) vs. T1 (18-22 ^o^C outdoor): 1.01 (0.68, 1.49) Lightheadedness T3 (30-33 ^o^C outdoor) vs. T1 (18-22 ^o^C outdoor): 1.36 (0.92, 2.01) Loss of consciousness T2 (30-33 ^o^C outdoor) vs. T1 (18-22 ^o^C outdoor): 0.62 (0.22, 1.73) Loss of consciousness T3 (30-33 ^o^C outdoor) vs. T1 (18-22 ^o^C outdoor): 1.78 (0.78, 4.07) Nausea T2 (30-33 ^o^C outdoor) vs. T1 (18-22 ^o^C outdoor): 1.28 (0.58, 2.81) Nausea T3 (30-33 ^o^C outdoor) vs. T1 (18-22 ^o^C outdoor): 2.57 (1.30, 5.06) Less frequent urination T2 (30-33 ^o^C outdoor) vs. T1 (18-22 ^o^C outdoor): 2.35 (1.11, 4.97) Less frequent urination T3 (30-33 ^o^C outdoor) vs. T1 (18-22 ^o^C outdoor): 3.65 (1.82, 7.35) Dark urine T2 (30-33 ^o^C outdoor) vs. T1 (18-22 ^o^C outdoor): 2.24 (1.39, 3.59) Dark urine T3 (30-33 ^o^C outdoor) vs. T1 (18-22 ^o^C outdoor): 1.92 (1.18, 3.12) Anxiety T2 (30-33 ^o^C outdoor) vs. T1 (18-22 ^o^C outdoor): 1.35 (0.87, 2.08) Anxiety T3 (30-33 ^o^C outdoor) vs. T1 (18-22 ^o^C outdoor): 1.50 (1.01, 2.23) Depressive symptoms T2 (30-33 ^o^C outdoor) vs. T1 (18-22 ^o^C outdoor): 1.56 (0.97, 2.51) Depressive symptoms T3 (30-33 ^o^C outdoor) vs. T1 (18-22 ^o^C outdoor): 1.28 (0.81, 2.04) Thirst T2 (30-33 ^o^C outdoor) vs. T1 (18-22 ^o^C outdoor): 2.00 (1.47, 2.73) Thirst T3 (30-33 ^o^C outdoor) vs. T1 (18-22 ^o^C outdoor): 3.39 (2.54, 4.52) |
| Uejio, 2016 | To determine whether cardiovascular or respiratory cases experience hotter indoor conditions as compared to controls. | Distress calls related to respiratory cases and cardiovascular cases | 764 total participants. Men and women (44%), All ages were included, ages ranged from 0-98 years old. | Indoor temperature (degree Celsius) (type undefined) was measured using HOBO sensors. | Case-control study, Generalized linear models, Models adjusted for time of day and air pollution (ozone and PM_2.5_) . | Odds ratio and 95% CI for distress calls and indoor temperatures (°C) above 26°C  Respiratory calls for changes of indoor temperature >26 °C: 1.43 (0.97, 2.10)  Cardiovascular calls for changes of indoor temperature >26 °C: 0.85 (0.55, 1.31) |
| Uejio, 2022 | To estimate the associations between indoor heat exposure and exacerbations of respiratory illness and diabetes that are reflected through 9-1-1 call incidences and patient care reports. | Respiratory illness and diabetes | 698 total participants. Men and women, All ages included, ages ranged from 0-100 | Indoor temperature (degree Celsius) (type undefined) was measured using HOBO sensors. | Case-control study, Generalized additive models, linear models, Models adjusted for time of day and air pollution (ozone and PM2.5 um). | Odds ratio and 95% CI for 9-11 emergency medical dispatch and services documentation and indoor temperatures (°C)  Diabetes calls for changes of indoor temperature from 30°C to 31°C: 1.12 (1.08,1.16)  Respiratory calls for changes of indoor temperature from 34 °C to 35°C: 1.18 (1.09, 1.28) |
| van Loenhout, 2016 | To investigate the relationship between indoor and outdoor temperature and heat-related health problems of elderly individuals during the summer months in the Netherlands | Annoyance by heat, breathing discomfort, fatigue, annoyance by heat at night and sleep disturbance | 113 total participants. Men and Women, older population (mean age 73.8 yrs, SD=7.5) | Indoor air temperature as mean daily temperature were measured (degrees Celsius) using iButton Hygrochron temperature/humidity sensors, (type DS1923; Maxim Integrated, San Jose, CA, USA). | Cross-sectional, Generalized Estimated Equation (GEE) Poisson log-linear models assuming exchangeable correlation structures, accounting for clustering of observations within individuals, Models adjusted for age and sex | Risk ratios and 95%confidence intervals for heat discomfort outcomes and indoor temperature (daily mean temp for every 1 °C increase)  annoyed by heat in the living room (# hours): 1.33 (1.20, 1.48) breathing discomfort in the living room (# hours): 1.28 (1.13,1.45) fatigue due to heat in the living room (# hours): 1.30 (1.13, 1.50) annoyed by heat in the bedroom at night (# hours): 1.28 (1.17, 1.41) sleep disturbance in the bedroom at night (# hours): 1.24 (1.10, 1.40 ) |
| Vellei, 2017 | To measure associations between overheating patterns in vulnerable (social homes) versus non-vulnerable households. | Thermal comfort (overheating risk, perception of heat discomfort) | 55 households. Men and women, all ages | Indoor air temperature was measured (degrees Celsius) using DS 18B20 sensors. Authors employed data processing to ensure that indoor temperatures were not influenced by other factors such as radiators and/or other heating sources, and outside solar radiation  The authors analyzed indoor temperature as dT which was the difference between obtained room temperature and comfort temperature using the following equation  : dT = T_room_ − T_comf_ where T_comf_ = 0.33∗T_rm_ + 18.8 | Cross-sectional study. Logistic regressions were fitted using thermal preference (TPV), thermal sensation (TSV) and thermal acceptability votes (TAV) and calculated dT | Logistic regression for thermal preference and thermal sensation vote are statistically significant (p = 0.026 and 0.03 respectively), while the logistic regression for thermal acceptability vote did not reach statistical significance (p = 0.124). |
| Wei, 2022 | To investigate thermal adaptability introduced by the seasonal climate, and to investigate the seasonal thermal sensation, thermal acceptability, and thermal preference, and to create seasonal thermal adaptation models and thermal comfort zones for the studied subjects. | Thermal comfort (thermal acceptability, thermal sensation, thermal preference). | Spring months: 526 participants, Summer months: 609 participants, Men and women. The mean age for the spring subset was 24.2 years, and the mean age of the summer subset was 23.8 years. | Indoor temperature was measured  using thermal comfort data logger (1221, Denmark TR-72U). | Cross-sectional study. Linear regressions, stratified by season | Beta coefficients extracted from regression equation for thermal sensation vote and indoor temperature ( per 0.5 °C degree increase in temp)  Full population, spring subset  Thermal sensation vote: 0.043  Full population, summer subset  Thermal sensation vote: 0.314  Beta coefficients associated with Thermal acceptance and indoor temperature ( per 1 °C degree increase in temp) extracted from manuscript Figure 7  Full population, spring subset  Thermal acceptance ( percentage dissatisfied):0.006  Full population, summer subset  Thermal acceptance ( percentage dissatisfied):0.008 |
| Williams, 2019 | Estimate associations between indoor temperature during an extreme heat event and personal physiological responses to heat | Mean galvanic skin response and mean hourly heart rate. | 51 total participants. Men and women, mean age 65 years | Undisclosed monitors were used to measure indoor dry-bulb temperature, taken at hourly increments. | Cross-sectional  Generalized additive mixed models  Adjustments for building | The results indicated that the maximum hourly indoor temperature was a significant predictor (p < 0.001) of mean hourly heart rate after adjusting for building factors, and it showed a non-linear relationship. An optimal temperature for heart rate and galvanic skin response was found to be around 24°C. |
| Yadeta, 2022 | To examine the residential building occupants' thermal comfort status. | Thermal comfort | 430 total participants. Men and women, 18 years and older | Indoor temperature was measured (type undefined) in degrees Celsius using  a handheld measurements "AcuRite" digital thermometer. | Cross-sectional study, linear regression. | Beta coefficient extracted from regression equation for thermal sensation and indoor temperature (for every 1 °C degree increase): 0.171 (SE=0.01) |
| Yang, 2022 | To investigate what are the similarities or differences in indoor thermal environments among cities, towns, and rural residences. | Thermal sensation vote | 141 total participants. Men and women, all ages. | Indoor temperature was measured in degrees Celsius using  using the TH22R-EX | Cross-sectional study, linear regressions. | Beta coefficient extracted from regression equation for thermal sensation and indoor temperature (for every 1 °C degree increase) as stratified by location  city: 0.3043  town: 0.239  rural: 0.199 |
| Zhang, 2018 | To determine the relationship between indoor temperature and thermal sensation vote while awake and asleep based on subjective questionnaires and objective measurements. | Sleep and wakefulness | Indoor temperature measurement tool was undefined | Cross-sectional | Linear regression models, no adjustments. | As indoor operative temperature increased, thermal sensation vote while participants were awake and asleep also increased. Beta coefficient extracted from regression equation for thermal sensation and indoor temperature (for every 1 °C degree increase)    Sleep: 0.06  Wakefulness: 0.16 |
| Abbreviations  OR: Odds ratio  RR: Relative Risk  ER: Excess Risk  SE: standard Error  CI: Confidence Interval  °C = degrees Celsius  °F = degrees Fahrenheit  mmHg = millimeters of mercury | | | | | | |
